# HIV prevention where it is needed most: comparison of strategies for the geographical allocation of interventions

**DOI:** 10.1002/jia2.25020

**Published:** 2017-12-08

**Authors:** Sarah‐Jane Anderson, Peter D Ghys, Regina Ombam, Timothy B Hallett

**Affiliations:** ^1^ Department of Infectious Disease Epidemiology Imperial College London London UK; ^2^ UNAIDS Geneva Switzerland; ^3^ National AIDS Control Council Nairobi Kenya

**Keywords:** geographical prioritization, epidemiology, mathematical modelling, health policy, HIV prevention, resource allocation

## Abstract

**Introduction:**

A strategic approach to the application of HIV prevention interventions is a core component of the UNAIDS Fast Track strategy to end the HIV epidemic by 2030. Central to these plans is a focus on high‐prevalence geographies, in a bid to target resources to those in greatest need and maximize the reduction in new infections. Whilst this idea of geographical prioritization has the potential to improve efficiency, it is unclear how it should be implemented in practice. There are a range of prevention interventions which can be applied differentially across risk groups and locations, making allocation decisions complex. Here, we use mathematical modelling to compare the impact (infections averted) of a number of different approaches to the implementation of geographical prioritization of prevention interventions, similar to those emerging in policy and practice, across a range of prevention budgets.

**Methods:**

We use geographically specific mathematical models of the epidemic and response in 48 counties and major cities of Kenya to project the impact of the different geographical prioritization approaches. We compare the geographical allocation strategies with a nationally uniform approach under which the same interventions must be applied across all modelled locations.

**Results:**

We find that the most extreme geographical prioritization strategy, which focuses resources exclusively to high‐prevalence locations, may substantially restrict impact (41% fewer infections averted) compared to a nationally uniform approach, as opportunities for highly effective interventions for high‐risk populations in lower‐prevalence areas are missed. Other geographical allocation approaches, which intensify efforts in higher‐prevalence areas whilst maintaining a minimum package of cost‐effective interventions everywhere, consistently improve impact at all budget levels. Such strategies balance the need for greater investment in locations with the largest epidemics whilst ensuring higher‐risk groups in lower‐priority locations are provided with cost‐effective interventions.

**Conclusions:**

Our findings serve as a warning to not be too selective in the application of prevention strategies. Further research is needed to understand how decision‐makers can find the right balance between the choice of interventions, focus on high‐risk populations, and geographical targeting to ensure the greatest impact of HIV prevention.

## 1 Introduction

Geographically specific intervention programming is commonly used in the control of a number of major infectious diseases, most notably those caused by vector‐borne agents such as malaria or schistosomiasis 1, 2. Application of such a strategy to HIV prevention is increasingly gaining support and has the potential to improve efficiency and maximize the impact of future programmes 3. There is substantial evidence of significant heterogeneity in the intensity of the epidemic, its key drivers, and the success of the response, not just between regions or countries but at local levels – between subnational divisions, towns and communities 4, 5, 6, 7. The greater understanding of the geographical diversity in the epidemic creates the opportunity to be increasingly strategic in the application of interventions for the prevention of transmission both through differential intensification across regions and through ensuring that strategies are suitable for the epidemic dynamics in the local area. Such a strategy will be central to the renewed focus on improving the efficiency of prevention investments through ensuring programmes reach those in greatest need, and is a cornerstone of the UNAIDS (Joint United Nations Programme on HIV/AIDS) Fast Track strategy to end the epidemic 8, 9.

The question now arises how such geographical targeting should be applied in practice with a number of different approaches seen in existing programmes. The most established approach for the differential allocation of intervention funds across regions is a “formula funding” strategy, whereby resources are divided between geographical areas in proportion to key indicators in each region (such as the number of PLHIV (people living with HIV), the number of people on treatment, or broader health systems, demographic or development measures) 10. Such strategies have been applied extensively in a range of countries providing a transparent means of allocating central funds 11. At the national level, many countries “classify” their subnational regions according to the differences in the intensity and type of epidemic seen, to allow for specific guidance based on the type of epidemic in each location. For example, India developed a four‐level classification system across districts with different interventions utilized in each district category 12, 13. Similarly, the Kenyan National AIDS programme has developed the Prevention Revolution strategy 14, which assigns counties to “clusters” based on HIV prevalence and recommends packages of interventions specific to each cluster. At the international level, funders are increasingly seeking more strategic approaches to allocation of their resources both across and within countries. PEPFAR (The United States President's Emergency Plan for AIDS Relief) have announced their intention to focus on those subnational areas with the highest HIV prevalence and lowest treatment coverage in a bid to direct funding to where it can have greatest impact. Such a strategy represents more extreme geographical prioritization, through restricting funds to only a proportion of subnational locations based on the intensity of the epidemic. Whilst the need to improve treatment coverage in areas with low current levels is clear, there is a danger that prevention programming may also be applied only where there are intensified treatment operations, denying critical prevention interventions to lower‐priority areas. Recent reports of their withdrawal of VMMC (Voluntary Medical Male Circumcision) services in some areas of Zimbabwe, due to the relatively low priority of these regions 15, have led to questioning of this approach.

With so many examples of geographically focused policies, it is critical that their respective approaches be rigorously evaluated. Here, we use a geographically specific model of HIV transmission of Kenya 3 to explore the cost and impact of different approaches to the geographical allocation of HIV prevention interventions. All of the geographical allocation strategies are compared with a “uniform” strategy under which all locations must receive the same set of interventions, to assess the value of the different approaches to geographical prioritization.

## 2 Methods

### 2.1 Mathematical models

This work utilizes county‐specific models of heterosexual and male‐to‐male transmission of HIV developed for Kenya with models tailored to reflect the differences in the epidemic and response across locations 3. Forty‐eight different locations were modelled: corresponding to the 47 counties of Kenya, with Kisumu County divided into urban and rural areas to allow for inclusion of additional Kisumu City data. Where available, models were informed by location‐specific data (Supporting Information).

### 2.2 Analytical approach

We sought to understand how “geographical prioritization” of HIV prevention can be applied with generalizable allocation policies and whether such strategies may improve the impact of prevention programmes. We compare different approaches to the allocation of interventions across modelled locations (outlined in Table 1), and compare their modelled impact (i.e. infections averted) across a range of prevention budgets. These were also compared with a “uniform” approach in which the same interventions must be applied in all locations (i.e. no geographical prioritization).

**Table 1:** The key features of each allocation strategy

	Optimized geographically focused strategy	Formula‐based approach	Extreme geographical prioritization	Staggered implementation approach	Uniform approach
Outline of the strategy	Finds the optimal configuration of different intervention modalities across population groups and individual locations which maximizes the number of infections averted	Funds are divided between individual locations proportional to the number of PLHIV in each location. The intervention which can be implemented for the available funds in each location is chosen from the defined order of roll out of interventions (described in Section 2.4)	All interventions in the predefined order of roll out are provided to the locations in the highest prevalence category, before moving to progressively lower‐prevalence categories	Under this approach, even low‐prevalence areas receive a minimum package of intervention strategies, although intervention is consistently most intense in high‐prevalence areas (i.e. further along the predefined order of roll out)	National strategy i.e. all modelled locations must receive the same set of interventions and there is no tailoring of intervention choices across locations
Geographical unit of allocation	Individual location (each subnational unit can receive a different set of interventions)	Individual location (each subnational unit can receive a different set of interventions)	Locations are classified into categories based on HIV prevalence. Allocation is based on the prevalence category of the location	Locations are classified into categories based on HIV prevalence. Allocation is based on the prevalence category of the location	All locations must receive the same interventions (national strategy)
Order of roll out of intervention modalities by population group	Finds the optimal order of roll out of interventions across populations and locations (i.e. not predefined)	Uses a predefined order of intervention roll out of modalities by population group (described in Section 2.4)	Uses a predefined order of intervention roll out of modalities by population group (described in Section 2.4)	Uses a predefined order of intervention roll out of modalities by population group (described in Section 2.4)	Uses a predefined order of intervention roll out of modalities by population group (described in Section 2.4)

PLHIV, people living with HIV.

A range of different intervention modalities are included in the prevention strategies: accelerated access to ART (antiretroviral therapy), behaviour change (BC), PrEP (pre‐exposure prophylaxis) and VMMC, which can be targeted by population group (heterosexual men, MSM (men who have sex with men), low‐risk women and FSW (female sex workers)). In all of the modelled strategies, we assume that treatment is available to everyone (i.e. all population groups and locations) with initiation at an average CD4 cell count of 200 cells per microlitre, corresponding to those actively seeking treatment. The “accelerated access to ART” prevention intervention is in addition to this background “late ART,” and represents active outreach to those with higher CD4 counts.

### 2.3 Comparison of policies for the allocation of interventions across locations

Key features of the different approaches to the allocation of interventions across locations are outlined in Table 1, with some strategies able to allocate interventions across individual locations and others across groups of locations (based on their prevalence category).

#### 2.3.1 Strategies with allocation by individual location

Previously, we developed an optimized “geographically focused” strategy for the design of combination prevention programmes, which strategically allocates resources across populations and geographies to maximize impact for a given budget 3. The optimized geographically focused strategy is able to allocate intervention modalities (accelerated access to ART, BC, PrEP and VMMC) differentially across population groups (heterosexual men, MSM, low‐risk women and FSW) and individual locations (counties and major cities of Kenya). The optimal configuration of interventions is identified which averts the greatest number of infections between 2015 and 2030 for a given prevention budget. Whilst this level of specificity in targeting improves impact, it may be challenging to implement in practice and relies upon a detailed representation of local epidemiology and complex optimization routines with a very large number of different possible intervention configurations.

The “formula‐based” approach is also able to allocate interventions individually across locations, but utilizes a predefined order of intervention roll out (discussed in Section 2.4). In this way, no optimization is required to construct this strategy, and it relies only on simple indicators (i.e. the number of PLHIV in each location). Under the formula‐based geographical strategy, funds are divided between individual locations proportional to the number of PLHIV in each location. The interventions adopted in each location are those possible for the available funds allocated to that location under the defined order of roll out.

#### 2.3.2 Strategies with allocation by categorized location

Under the “extreme geographical prioritization” and “staggered implementation” approaches, both locations and intervention choices are grouped to limit the number of allocation options and to make the strategies more transparent. We define location “prevalence” categories for the modelled locations and a standard order of roll out of different intervention modalities (discussed further in Section 2.4 below).

In the extreme geographical prioritization strategy, we present an extreme representation of geographical prioritization, whereby we provide all interventions to the locations in the highest prevalence category, before moving to progressively lower categories.

In the staggered implementation approach, we present a more moderate representation of geographical prioritization. Under this approach, even low‐prevalence areas receive intervention strategies (VMMC, BC for high‐risk people and accelerated access to ART), although intervention is consistently most intense in high‐prevalence areas. Whilst even low‐prevalence areas get the minimum intervention package, roll out of intervention strategies is always greatest in the higher‐prevalence locations.

We compare these geographical prioritization strategies with a uniform approach where all modelled locations must receive the same set of interventions and there is no tailoring of intervention choices across locations.

We assess the percentage difference in impact (i.e. number of infections averted compared to baseline projections in the absence of scale‐up of prevention programmes) between each geographical strategy and the uniform strategy at a range of budget levels (at $100 million intervals between $500 million to $30000 million over the 15‐year time period).

### 2.4 Definition of prevalence categories and the order of roll out of interventions

As we seek to provide a simplified representation of the policy choices, for the extreme geographical prioritization and staggered implementation approaches, we limit the number of locations across which we allocate interventions through grouping modelled counties into “very high,” “high,” “medium” and “low” prevalence categories using k‐means clustering.

In order to standardize the comparison between the allocation strategies, a predefined order of intervention roll out was defined for the simple geographical strategies (formula‐based, extreme geographical prioritization and staggered implementation) and uniform approach. We consider four different intervention modalities (PrEP, BC, accelerated ART and VMMC) which can be applied to high‐risk populations (FSW and MSM) or low‐risk populations (heterosexual men and low‐risk women) only, or to men or women only. The relative priority of the different interventions across population groups is given in Table 2, and reflects the cost‐effectiveness ratio of each intervention applied individually at national level. The next intervention stage can only be completed once the preceding interventions have been applied (i.e. interventions are additive at each step).

**Table 2:** The different interventions included in each stage of programme roll out

Priority for roll out^a^	Intervention	Efficacy assumption	Cost assumption	Coverage assumption (predefined target level)	Explanation
1	VMMC for those locations where roll out has not yet reached target levels	Risk of infection 60% less in circumcised men 16	$60 per procedure 17	80% of eligible men	Male circumcision is a highly favourable intervention as it is a “one‐off” cost, cheap and efficacious. Kenya historically had only a relatively small number of counties with low levels of circumcision and implementation of “VMMC” here refers to circumcising in those counties where coverage is not at target levels.
2	Behaviour change in high‐risk populations (FSW and MSM)	50% reduction in risk (assumed greater impact of BC in high‐risk groups)	$20 per person per year 17	100%^b^	Behaviour change in high‐risk populations is assumed to be relatively efficacious at a low cost.
3	Accelerated ART in all men	85% reduction in risk of transmission for a person on ART relative to others 18, 19, 20.	$515 per person per year 21	33% heterosexual men, 66% MSM	The recent ART guidelines released by the WHO recommend the provision of treatment to all those HIV‐positive irrespective of the stage of infection. Here, we consider accelerated scale‐up of ART in the population as priority for roll out. In the model, all individuals receive treatment at an average CD4 of 200. This “accelerated ART” intervention is in addition to this background “late” ART and is for those with higher CD4 counts, with active outreach to engage them in services. As women are at greater risk of acquiring infection than men, accelerated outreach of ART to men before women averts a greater number of infections through reducing onward transmission to women.
4	Accelerated ART in all women	85% reduction in risk of transmission for a person on ART relative to others 18, 19, 20.	$515 per person per year 21	33% low‐risk women, 66% FSW	
5	PrEP in high‐risk populations	75% reduction in risk of infection for a person on PrEP relative to others 21.	$250 per person per year 21	50%	PrEP is an effective, but expensive intervention. It is prioritized first to the highest risk populations.
6	Behaviour change in low‐risk populations	20% reduction in risk	$10 per person per year 17	100%^b^	Behaviour change in low‐risk populations is assumed to have only modest impact.
7	PrEP in low‐risk populations	75% reduction in risk of infection for a person on PrEP relative to others 21.	$250 per person per year 21	25%	PrEP is unlikely to be provided to low‐risk populations as it is a very expensive intervention.

ART, antiretroviral therapy; BC, behaviour change; MSM, men who have sex with men; FSW, female sex workers; WHO, World Health Organization; PrEP, pre‐exposure prophylaxis.

a The order reflects the individual cost‐effectiveness ratio from applying each intervention at national level.

b Here, the coverage corresponds to the efficacy of the intervention; as such a programme would be provided to the entire population but will only alter the behaviour of a proportion of the population.

## 3 Results

**Figure 1:**
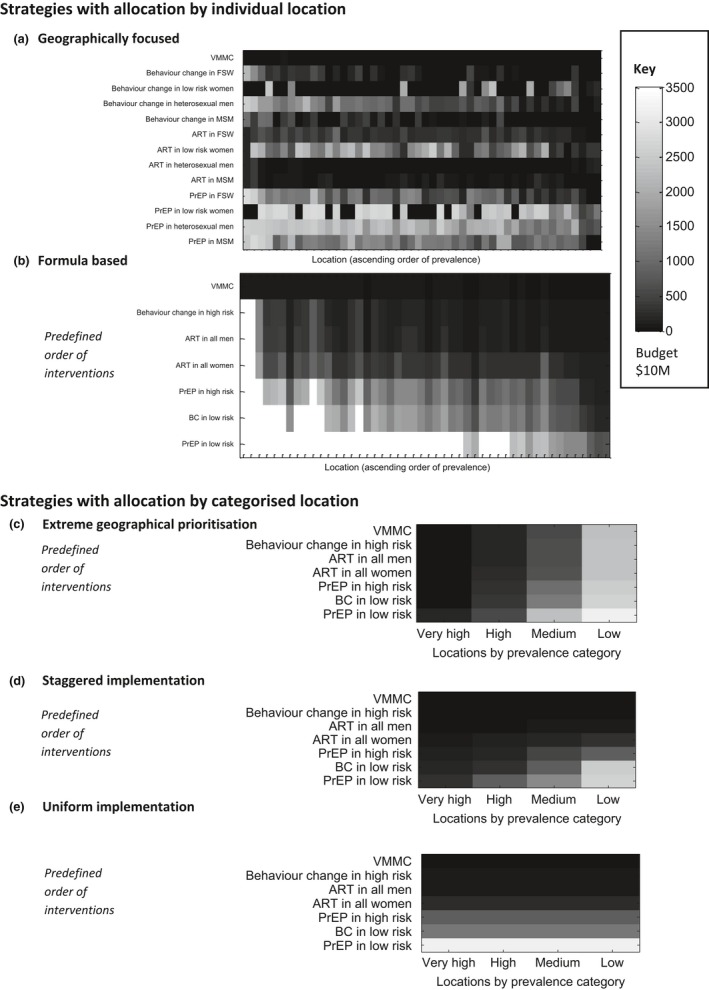
Roll out of interventions by allocation strategy. Each panel demonstrates the order of implementation of the different intervention strategies (vertical axis) across locations (horizontal axis) for each allocation strategy. Dark shades indicate a high‐priority intervention (implemented even with a low available budget) and light shades a lower‐priority intervention. The key gives the corresponding budget level at which a given intervention is implemented. Here, location refers to the modelled county or city. ART refers to an active outreach programme to accelerate treatment coverage in a given population or location. VMMC refers to voluntary medical male circumcision and PrEP refers to pre‐exposure prophylaxis. BC refers to behaviour change interventions. Further details of each intervention component are provided in Section 2.2.

**Figure 2:**
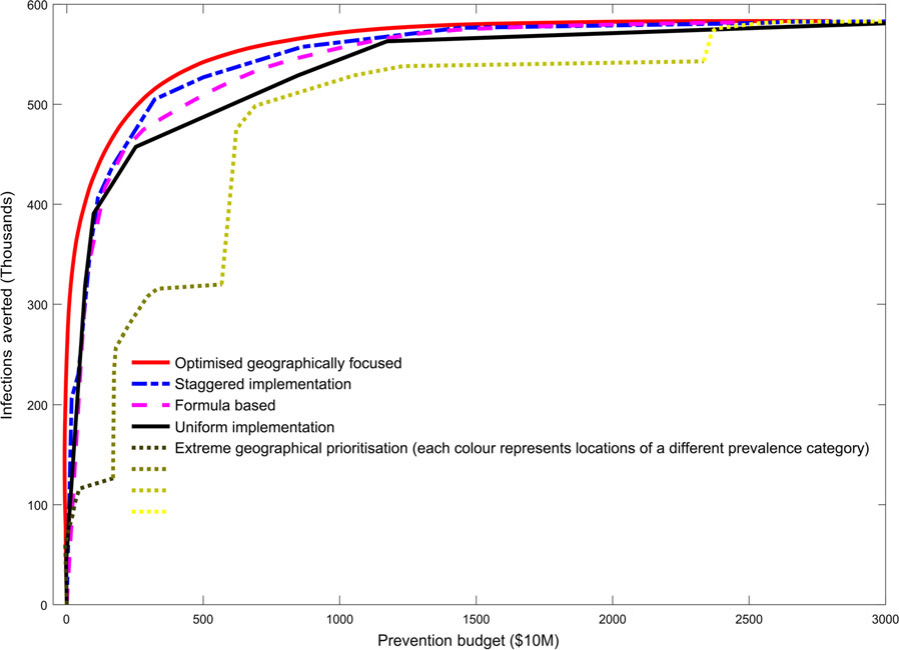
Impact of each allocation strategy. The modelled impact (infections averted 2015 to 2030) under each of the allocation strategies across a range of prevention budget levels.

We first examine the order of roll out of interventions across modelled locations under each of the strategies (Figure 1). Each panel of Figure 1 represents a different allocation strategy, with the intervention by population group (either applied individually or grouped dependent on the strategy) on the vertical axis and the location (either considered individually or within a prevalence category) on the horizontal axis. Those interventions which are shaded in dark colours are high priority (implemented even at low budgets). Those shaded in lighter colours are lower priority (implemented only at high budgets). We then compared the modelled impact (number of infections averted) under each strategy across different levels of spending (Figure 2).

### 3.1 The order of roll out of strategies

The optimized geographically focused (Figure 1a) approach has a complex order of roll out across interventions and locations with increasing levels of prevention spending. The order of roll out presented corresponds to the maximum possible impact of a geographically focused strategy (Section 2.3.1). The plot demonstrates that some interventions are more readily used than others, and consistently implemented even at low budgets (e.g. BC in FSW), whilst others are less favourable and are generally only applied at high budget levels (e.g. PrEP in low‐risk women). Whilst it is possible to draw some conclusions about the relative importance of different interventions, there is a strong interaction with the location, meaning some locations receive a greater number of intervention strategies.

The formula‐based (Figure 1b) approach relies only on simple indicators (i.e. the number of PLHIV in each location) and a predefined order of roll out of interventions. The allocation is constrained to follow the defined order of roll out (vertical axis) but is applied at different intensities across locations (horizontal axis) according to the number of PLHIV in each area.

The extreme geographical prioritization strategy (Figure 1c) concentrates funds exclusively on the highest prevalence locations at low budgets. This strategy only expands to lower‐prevalence locations following complete roll out of all interventions in higher‐prevalence locations first.

Under the staggered implementation approach (Figure 1d), even low‐prevalence areas receive a minimum package of intervention strategies (VMMC, BC for people at high risk and accelerated access to ART (i.e. active outreach) i.e. those most favourable in the predefined order of roll out), although intervention is consistently most intense in high‐prevalence areas.

Under the uniform approach (Figure 1e), all locations must receive the same set of interventions. The order in which interventions are included as budgets become larger follows the predefined order of roll out (vertical axes). This scenario serves as the comparison to the geographical strategies defined and allows for evaluation of the value of the different approaches to geographical prioritization.

### 3.2 The potential impact of each strategy

The strategies vary substantially in how effectively they avert infections (Figure 2), with considerable differences observed at some prevention budget levels. Indeed, this difference in impact between the geographical allocation strategies is most pronounced at moderate budgets, encompassing the projected resources available for HIV prevention in Kenya 22. The impact of each geographical allocation strategy is considered relative to the uniform approach.

The optimized geographically focused approach has complete flexibility in allocation and finds the optimal order of roll out with the greatest impact at all levels of spending (as much as 19% more of future infections averted relative to the uniform approach under the budget levels considered).

The staggered implementation strategy performed consistently stronger than the uniform approach across all budget levels, with up to 10% more infections are averted for the same budget at moderate levels of prevention spending. This demonstrates that higher‐intensity implementation in the high‐priority locations is beneficial whilst maintaining a minimum package of interventions across all locations. It is unable to perform as strongly as the original optimized geographically focused approach as it is unable to be as targeted, with the order of roll out of interventions fixed for all locations.

The formula‐based approach is able to generate marginal gains over the uniform approach (up to 5% under the budget levels considered). The number of PLHIV, although a useful indication of epidemic intensity, is not a direct measure of prevention need or the effect these can have on local dynamics, and so it is unable to perform as strongly.

The extreme geographical prioritization strategy performs substantially less well than the uniform strategy, and may lead to less health impact than if interventions are applied uniformly across the country. The loss of impact could be much as 41% at moderate budget levels. The “jumps” in impact observed across budgets represent the change from a low‐priority intervention strategy in a higher‐prevalence category, to a higher priority intervention in a lower‐prevalence category (the different shades in Figure 2). The limitations of this approach stem from the requirement to apply low‐priority intervention modalities in high‐prevalence locations before the programme can be expanded to high‐risk populations elsewhere.

## 4 Discussion

Prioritizing prevention interventions to those in greatest need will be central to maximizing the impact of programmes and ensuring progress towards ambitious HIV prevention goals. However, HIV programmes are complex and composed of different prevention modalities applied differentially across population groups and locations. A focus on geography alone in the allocation of resources may miss opportunities for application of highly effective interventions elsewhere or a focus on high‐risk population groups. Using stylized examples, we demonstrate that whilst geographical prioritization could be a useful tool for improving the impact of HIV prevention programmes, it also has the potential to be detrimental.

The extreme geographical prioritization strategy, designed to reflect restriction of investment in lower‐prevalence geographies to allow for intensification in high‐prevalence areas (similar to PEPFAR's approach), is found to be the least impactful at country level. Our analysis shows that limiting investment to those locations with the most intense epidemics in this way may significantly reduce impact (41% fewer infections averted at moderate budget levels) compared to a nationally uniform approach with no geographical prioritization. This strategy misses the opportunity to avert infections across the remainder of locations in the country, particularly in high‐risk populations, as it devotes resources to lower‐priority interventions in a small number of the highest priority locations. Such extreme geographical prioritization therefore restricts the number of infections it is possible to avert. Furthermore, such a strategy will have important ethical and political consequences. Often lower‐prevalence areas are rural and underserved, and require greater investment to maintain basic services. The absence of services may give the misleading signal that they are not required, even though, by international standards, HIV remains a significant public health problem.

Provisions must therefore be made to ensure that essential highly cost‐effective prevention services are maintained across all settings. Country governments must ensure areas without donor funds still receive sufficient support for prevention services. Similar to that presented in the staggered implementation approach here, a “minimum package” of the most cost‐effective interventions is needed across all settings. In this way, some interventions are so favourable they are exempt from geographical prioritization, including VMMC, which as a cheap, highly effective and lifelong intervention, is one of the most cost‐effective prevention interventions available at this time. In those settings which have not yet reached VMMC targets, further expansion should be an immediate priority. Further research is needed to explore barriers to achieving target coverage for VMMC, particularly understanding factors influencing demand for VMMC services across populations. The staggered implementation strategy has many similarities to the cluster‐based approaches of India and Kenya 14.

Similarly, the formula‐based approach, through dividing funds proportional to the number of PLHIV in each location, ensures that most locations receive some essential services. Whilst this approach considers only one indicator (PLHIV), other formula‐based approaches may use multiple indicators, often not specific to HIV funding or the healthcare sector, undermining the ability to allocate funds based on the heterogeneity of the epidemic. Even with simple HIV indicators including the number of PLHIV, such approaches do not fully capture the differential opportunities for intervention.

To fully maximize the impact of “geographical prioritization,” we require an understanding of the patterns of transmission between risk groups in each setting to find the optimal choice of intervention by population and location. A number of modelling tools (including Optima 23, GOALS 24 and AEM 25), used to support national and international decision‐making, are increasingly being applied at subnational level. These models aid in the design of prevention programmes similar to the optimized geographically focused strategy presented here. The use of these tools for geographical prioritization, taking into account all expected donor and national resources, needs further exploration.

A number of extensions to this analysis could be explored. Examination of the impact of these strategies under different prevalence category definitions, target coverage levels or order of intervention roll out may allow for greater impact. Further work could also look at different epidemic contexts, at national and international level. A number of other factors not included in this study will influence the outcome of geographical targeting. The cost of providing services will differ between locations, as is the case for providing treatment to PLHIV 26, 27 and in VMMC delivery 28. Furthermore, the unit costs of interventions are likely to be associated with the scale of services 29 and other factors such as synergies across different services. Econometric functions could be used to explore candidate relationships between the cost and scale of programmes. This study does not consider the costs associated with the complex changes in healthcare organization and funding provision inherent in geographical targeting, including the redistribution of financial and human resources and the development of new infrastructure. In practice, uncertainty in local epidemic data is an important limitation, and the value of collecting additional information to improve decision‐making must be assessed. Further work could also explore the implications of different approaches to prioritizing outreach for treatment and engaging HIV‐positive individuals in care. In this analysis, all interventions including active outreach to treatment can be targeted specifically by population group. However, it must be noted that active outreach for ART is in addition to the ART provided to all at low CD4 counts. Differentiating by population groups in this way allows for greater infections averted, whilst also ensuring all those in need will receive treatment. The current representation of treatment here assumes there will be a need to balance treatment outreach with other prevention interventions. As budgets become large enough however, active outreach to treatment programmes is used in all locations (resulting in almost 80% coverage at the end of the intervention period), in line with recent WHO (World Health Organization) guidelines 30.

## 5 Conclusions

Geographical prioritization is an important way of designing more effective and targeted prevention programmes but must be applied with caution. If funding is withdrawn from lower‐priority locations, it is critical that priority interventions are still maintained in these settings. For greatest impact, there needs to be a balance between choosing highly cost‐effective intervention modalities and strategic targeting to both priority populations and locations.

## Competing interests

SJA reports personal fees from The Bill & Melinda Gates Foundation, personal fees from Avenir Health, personal fees from Anansi Health and personal fees from the Global Fund outside the submitted work. TBH received grants and personal fees from the Bill & Melinda Gates Foundation during the conduct of the study; grants and personal fees from World Bank; grants from UNAIDS and The Rush Foundation; and personal fees from the University of Washington, New York University and Global Fund outside of the submitted work. For the remaining authors, none were declared.

## Authors’ contributions

SJA and TBH conceived the study and developed the methods and analysis. SJA wrote the first draft of the manuscript. PDG and RO advised on the interpretation of the analysis. All authors contributed to the writing of the manuscript and reviewed and approved the final version.

## Funding

Funding for this study was provided by the Bill & Melinda Gates Foundation OPP1084364 through a grant for the HIV Modelling Consortium to Imperial College London.

## Supporting information


**Data S1.** Mathematical models.
