# Supplementary material for: HIV prevention where it is needed most: comparison of strategies for the geographical allocation of interventions
Source: J Int AIDS Soc. 2017 Dec 8;20(4):e25020. doi: 10.1002/jia2.25020 (PMC5810320; doi:10.1002/jia2.25020)
Supplement: Supplementary file 1 — Data S1. Mathematical models. [file JIA2-20-e25020-s001.docx]

**Supplementary Information**

The mathematical models used in this analysis have been described elsewhere [1]. Following others, the mathematical models describe transmission of HIV in the population, and progression through the different stages of infection [2-4].

## Description of the Model

The model is described in nine sections: 1 Differential Equations, 2 The Natural History of Infection, 3 Demography, 4 The Definition of Risk Groups, 5 The Incorporation of Interventions, 6 The Calculation of the Force of Infection, 7 Model Fitting, 8 Input Parameter Tables and 9 Methods for the Interpolated Prevalence Maps.

### Differential Equations

The model is described by a set of ordinary differential equations solved numerically using Euler’s Method. The counties and three major cities of Kenya (Nairobi, Mombasa, and Kisumu) are modelled independently (denoted as location l). Each county or city specific model is tailored to reflect the estimated mapped HIV prevalence and key indicators including circumcision rates and reported sexual behaviour. The sexually active population (15-49, denoted a=1) is stratified by both sex and partner preference (k=1 heterosexual females, k=2 heterosexual males, and k=3 MSM) and reported risk behaviour (denoted as r). The model also distinguishes between those who receive PrEP and circumcision status in men and those who receive PrEP in women (intervention status denoted i). These stratifications are discussed further in Section 5. ART use and death are also described in the population aged 50+ (a= 2) however no further transmission is considered in this group.

The natural history of HIV infection and treatment is described by eight states (s), HIV negative${( X}^{1}$), HIV positive acute stage${( X}^{2}$), HIV positive with CD4>500${( X}^{3}$), HIV positive with CD4 350-500${( X}^{4}$), HIV positive with CD4 200-350 ($X^{5}),$HIV positive with CD4 <200${( X}^{6}$), HIV positive with CD4<200 prior to ART initiation${( X}^{7}$) and HIV positive on ART${( X}^{8}$).

The ordinary differential equations below describe the movement between these states in the population aged 15-49 (a=1). The state variables are of the form $X_{r,i,a}^{s,l,k}.$

**In susceptible (HIV negative) individuals:**

**In women (k=1):**

$\frac{{dX}_{r,i,1}^{1,l,1}}{dt}= E_{r,i}^{l,1}-\left( \lambda_{r,i}^{l,1}+\mu_{1} \right)X_{r,i,1}^{1,l,1}$

**In men (k=2, k=3):**

$\frac{{dX}_{r,1,1}^{1,l,k}}{dt}= E_{r,1}^{l,k}-(\lambda_{r,1}^{l,k}+\mu_{1})X_{r,1,1}^{1,l,k}{-n}_{c}X_{r,1,1}^{1,l,k}$

$\frac{{dX}_{r,2,1}^{1,l,k}}{dt}= E_{r,2}^{l,k}-\left( \lambda_{r,2}^{l,k}+\mu_{1} \right)X_{r,2,1}^{1,l,k}{+n}_{c}X_{r,1,1}^{1,l,k}$

$\frac{{dX}_{r,3,1}^{1,l,k}}{dt}= E_{r,3}^{l,k}-\left( \lambda_{r,3}^{l,k}+\mu_{1} \right)X_{r,3,1}^{1,l,k}{-n}_{c}X_{r,3,1}^{1,l,k}$

$\frac{{dX}_{r,4,1}^{1,l,k}}{dt}= E_{r,4}^{l,k}-(\lambda_{r,4}^{l,k}+\mu_{1})X_{r,4,1}^{1,l,k}{+n}_{c}X_{r,3,1}^{1,l,k}$

**In those HIV positive:**

$\frac{{dX}_{r,i,1}^{2,l,k}}{dt}={\lambda_{r,i}^{l,k}X}_{r,i,1}^{1,l,k}-(\mu_{1}+\sigma_{2})X_{r,i,1}^{2,l,k}$

$\frac{{dX}_{r,i,1}^{3,l,k}}{dt}=\gamma_{3}{\sigma_{2}X}_{r,i,1}^{2,l,k}-{\left( \mu_{1}+\sigma_{3}+\omega_{r,3,l,k} \right)X}_{r,i,1}^{3,l,k}$

$\frac{{dX}_{r,i,1}^{4,l,k}}{dt}={{\gamma_{4}{\sigma_{2}X}_{r,i,1}^{2,l,k}+\sigma}_{3}X}_{r,i,1}^{3,l,k}-(\mu_{1}+\sigma_{4}+\omega_{r,4,l,k})X_{r,i,1}^{4,l,k}$

$\frac{{dX}_{r,i,1}^{5,l,k}}{dt}={{\gamma_{5}{\sigma_{2}X}_{r,i,1}^{2,l,k}+\sigma}_{4}X}_{r,i,1}^{4,l,k}-{(\mu_{1}+\sigma_{5}+\omega_{r,5,l,k})X}_{r,i,1}^{5,l,k}$

$\frac{{dX}_{r,i,1}^{6,l,k}}{dt}=(1-a^{l}(t))({{\gamma_{6}{\sigma_{2}X}_{r,i,1}^{2,l,k}+\sigma}_{5}X}_{r,i,1}^{5,l,k})-{(\mu_{1}+\sigma_{6}+\omega_{r,6,l,k})X}_{r,i,1}^{6,l,k}$

$\frac{{dX}_{r,i,1}^{7,l,k}}{dt}=a^{l}(t)({{\gamma_{6}{\sigma_{2}X}_{r,i,1}^{2,l,k}+\sigma}_{5}X}_{r,i,1}^{5,l,k})-{(\mu_{1}+\sigma_{7}+\omega_{r,7,l,k})X}_{r,i,1}^{7,l,k}$

$\frac{{dX}_{r,i,1}^{8,l,k}}{dt}={\sigma_{7}X}_{r,i,1}^{7,l,k}+({\omega_{r,3,l,k}X}_{r,i,1}^{3,l,k}+\omega_{r,4,l,k}X_{r,i,1}^{4,l,k}+{\omega_{r,5,l,k}X}_{r,i,1}^{5,l,k}+{\omega_{r,6,l,k}X}_{r,i,1}^{6,l,k}+{\omega_{r,7,l,k}X}_{r,i,1}^{7,l,k})-{(\mu_{1}+\sigma_{8})X}_{r,i,1}^{8l,k}$

Where $E_{r,i}^{l,k}$ is the number of individuals entering the population group (r,i,k) in location l , $n_{c}$ the rate of circumcision in susceptible men, $\gamma_{s}$ the proportion of those leaving the acute stage who immediately enter each of the later stages, $\lambda_{r,i}^{l,k}$ the force of infection, $\mu_{1}$ the rate of aging from the 15-49 group, $\sigma_{s}$ the rate of progression between states, $a^{l}(t)$ the ART coverage for those receiving late ART and $\omega_{r,s,l,k}$ the rate of accelerated ART. Further details about the calculation and resulting values assigned to each of these parameters are given in later sections.

In the population aged 50+ the equations are as below. As no transmission is considered in this age group, intervention, risk and partner preference groups from the younger age group are amalgamated, with the state variable of the form$Y_{l}^{s}$, where s corresponds to the natural history state and l the location of interest.

$\frac{{dY}_{l}^{1}}{dt}=\sum_{r} \sum_{i} \sum_{k} X_{r,i,1}^{1,l,k}\mu_{1}-\mu_{2}Y_{l}^{1}$

$\frac{{dY}_{l}^{2}}{dt}=\sum_{r} \sum_{i} \sum_{k} X_{r,i,1}^{2,l,k}\mu_{1}-(\mu_{2}+\sigma_{2})Y_{l}^{2}$

$\frac{{dY}_{l}^{3}}{dt}=\sum_{r} \sum_{i} \sum_{k} X_{r,i,1}^{3,l,k}\mu_{1}+{\sigma_{2}Y}_{l}^{2}-{{(\mu}_{2}+\sigma_{3})Y}_{l}^{3}$

$\frac{{dY}_{l}^{4}}{dt}=\sum_{r} \sum_{i} \sum_{k} X_{r,i,1}^{4,l,k}\mu_{1}+\sigma_{3}Y_{l}^{3}-{(\mu}_{2}+\sigma_{4})Y_{l}^{4}$

$\frac{{dY}_{l}^{5}}{dt}=\sum_{r} \sum_{i} \sum_{k} X_{r,i,1}^{5,l,k}{\mu_{1}+\sigma}_{4}Y_{l}^{4}-(\mu_{2}{+\sigma_{5})Y}_{l}^{5}$

$\frac{{dY}_{l}^{6}}{dt}=\sum_{r} \sum_{i} \sum_{k} X_{r,i,1}^{6,l,k}{\mu_{1}+(1-a^{l}(t))\sigma}_{5}Y_{l}^{5}-{\left( \mu_{2}+\sigma_{6} \right)Y}_{l}^{6}$

$\frac{{dY}_{l}^{7}}{dt}=\sum_{r} \sum_{i} \sum_{k} X_{r,i,1}^{7,l,k}{\mu_{1}+a^{l}(t)\sigma}_{5}Y_{l}^{5}-(\mu_{2}+\sigma_{7})Y_{l}^{7}$

$\frac{{dY}_{l}^{8}}{dt}=\sum_{r} \sum_{i} \sum_{k} X_{r,i,1}^{8,l,k}{\mu_{1}+\sigma}_{7}Y_{l}^{7}-{(\mu}_{2}+\sigma_{8})Y_{l}^{8}$

Where $\sigma_{s}$ is the rate of progression between states, $a^{l}(t)$ the coverage of ART in location l,$\mu_{1}$ the rate of aging from the 15-49 population into the 50+ population, and $\mu_{2}$ the background rate of mortality in the population aged 50+. Interventions are only targeted to the sexually active (15-49) population and as such accelerated ART and circumcision are not considered in the 50+ population.

### The Natural History of Infection

Following the acute stage of infection, not all individuals pass through all CD4 states sequentially, with some entering later stages with lower CD4 counts immediately. Here the proportion of individuals who enter each state following acute stage is defined by the parameter $\gamma_{s}$(Table S1). No individuals enter the ‘on ART’ (s=8) state immediately after acute infection. The parameter $\sigma_{s}$ describes the rate of progression between infection states (Table S2).

Table S1. The proportion of individuals who enter each state following acute infection.

| Parameter | Movement from acute into category | Value | Reference |
| --- | --- | --- | --- |
| $\boldsymbol{\gamma}_{\boldsymbol{3}}$ | CD4>500 | 0.58 | Lodi et al 2011 [5] |
| $\boldsymbol{\gamma}_{\boldsymbol{4}}$ | CD4 350-500 | 0.23 | Lodi et al 2011 [5] |
| $\boldsymbol{\gamma}_{\boldsymbol{5}}$ | CD4 200-350 | 0.16 | Lodi et al 2011 [5] |
| $\boldsymbol{\gamma}_{\boldsymbol{6}}$ | CD4 <200 | 0.03 | Lodi et al 2011 [5] |

Table S2. The rate of progression between infection states.

| Parameter | Applied to State | Explanation | Value | Reference |
| --- | --- | --- | --- | --- |
| $\boldsymbol{\sigma}_{\boldsymbol{2}}$ | Acute | Inverse of the mean duration of the Acute stage | 12/9 | Hollingsworth et al 2008 [6] |
| $\boldsymbol{\sigma}_{\boldsymbol{3}}$ | CD4 >500 | Inverse of the mean duration of the CD4 >500 stage | 1/6.37 | Fit to Lodi et al 2011 [5] |
| $\boldsymbol{\sigma}_{\boldsymbol{4}}$ | CD4 350-500 | Inverse of the mean duration of the CD4 350-500 stage | 1/86 | Fit to Lodi et al 2011 [5] |
| $\boldsymbol{\sigma}_{\boldsymbol{5}}$ | CD4 200-350 | Inverse of the mean duration of the CD4 200-350 stage | 1/3.54 | Fit to Lodi et al 2011 [5] |
| $\boldsymbol{\sigma}_{\boldsymbol{6}}$ | CD4 <200 (will not receive ART) | Death rate in those CD4<200 | 1/3 | Fit to Lodi et al 2011 [5] |
| $\boldsymbol{\sigma}_{\boldsymbol{7}}$ | CD4 <200 (will receive ART) | Inverse of mean waiting time to ART initiation. | 1/0.5 | Assuming a mean waiting time of 0.5 years |
| $\boldsymbol{\sigma}_{\boldsymbol{8}}$ | Receiving ART | Death rate on ART | 1/20 | Assuming a net life expectancy of 20 years after ART initiation |

### Demography

The population size of each location was taken from the County Profile Report [7] or the MARPs geographic Mapping Report [8] and is based on the 2009 census data. Where county and city is not synonymous (Kisumu) the city population was removed from the county population, and the city and remaining county considered separately. We consider both HIV related mortality and the background aging (in the 15-49 population) and death rates (in the 50+ population). In the 15-49 population the rate of aging into the 50+ group$( \mu_{1})$ is 1/35. In the 50+ population the rate of death $(\mu_{2})$ is assumed to be 1/15.

The total number of individuals who enter the model at age 15 in each location l, denoted ($K_{l}),$is calculated as follows:

$$I_{1}^{l}=\sum_{r} \sum_{i} \sum_{k} \sum_{s} X_{r,i,1}^{s,l,k}$$

$$I_{2}^{l}=\sum_{s} X_{2}^{s,l}$$

$$K_{l}=\mu_{2}I_{2}^{l}+\alpha(I_{1}^{l}+I_{2}^{l})$$

Where$I_{1}^{l}$is the total number of individuals in the 15-49 population,$I_{2}^{l}$ is the total number of individuals in the 50+ population, $\mu_{2}$ is the death rate in the population aged 50+ and $\alpha$ the annual natural growth rate of the population assumed to be 3% based on data from the World Bank [9].

The total number of individuals entering the population$K_{l}$is divided between the relevant subgroups according to the assumed distribution of sex and partner preference (k), risk behaviour (r) and intervention status (i) in the population in location (l). All individuals who enter the model are assumed to be susceptible, that is we do not consider infections that were acquired prior to the age of 15. The number of individuals entering each subpopulation$E_{r,i}^{l,k}$is given by:

$$E_{r,i}^{l,k}=K_{l}\varphi_{l,k,r,i}$$

where $\varphi_{l,k,r,i}$ is the fraction of individuals entering the model in location l of the sex and partnership preference group k, risk group r and intervention group i. It is assumed that half of the population entering the model are female and half male, and that 3% of men are MSM. The division of the population into risk groups is described further in Section 4 below. The parameters describing the proportion of the population who are in each risk group are included in the model fitting process (Section 7). This is informed by the reported risk behaviour by province from the 2008/2009 DHS [10] which is given in Table S16 in Section 8. It is assumed that no one entering the modelled population is on PrEP. The male population is divided between those circumcised or not circumcised based on the baseline proportion of men circumcised in each location is denoted$F_{l}^{1}$. The baseline proportion of men circumcised in each location is also included in the model fitting process (Section 7), however it is strongly informed by the data from the County Profiles Report [7]. This parameter is described further in Table S20 in Section 8.

### The Definition of Risk Groups

We divide the female and heterosexual male populations into three risk groups, with the parameters used to define the fraction in each risk group based on the reported sexual behaviour from the DHS 2008/2009 [10] (described in Table S16 in Section 8). The highest risk group (risk group 1) is defined as those that report engaging in commercial sex in the past 12 months. The medium risk group (risk group 2) report non-marital or non-cohabiting partnerships in the past 12 months. The fraction of the population remaining is considered low risk (risk group 3). The proportion of individuals of location l and of sex and partnership preference k who are in risk group r ($\psi_{l,k,r})$ is defined as:

**For Women (k=1) and Heterosexual Men (k=2):**

Risk Group 1 (Commercial Sex (FSW or Client))

$$\psi_{l,k,1}=R_{l,k,2}R_{l,k,1}$$

Risk Group 2 (Casual Sex)

$$\psi_{l,k,2}=R_{l,k,2}(1-R_{l,k,1})$$

Risk Group 3

$$\psi_{l,k,3}=1-R_{l,k,2}$$

Where $\psi_{l,k,r}$ is the proportion of women (k=1) or heterosexual men (k=2) in risk group r, $R_{l,k,2}$ is the proportion of men and women who engage in casual sex and $R_{l,k,1}$of those who engage in casual sex the proportion who engage in commercial sex. Both $R_{l,k,2}$and $R_{l,k,1}$ are included in the model fitting process (Section 7).

We describe the behaviour of MSM in different locations through dividing the MSM population into three risk groups. High risk MSM are divided into two groups based on whether they form homosexual partnerships exclusively (risk group 1) or can form both heterosexual and homosexual partnerships (risk group 2). Risk group 3 MSM are assumed to be at lower risk with all individuals assumed to be bisexual.

In county level models only MSM in risk group 3 are included, as such all MSM are assumed to be both low risk and bisexual.

**For Counties:**

Risk Group 1 (High Risk Exclusive MSM)

$$\psi_{l,3,1}=0$$

Risk Group 2 (High Risk Bisexual)

$$\psi_{l,3,2}=0$$

Risk Group 3 (Low Risk Bisexual)

$$\psi_{l,3,1}=1$$

In city level models all three risk groups are included. The fraction of MSM $(\psi_{l,3,r})$ in each risk group r in city level models is calculated as follows:

**For Cities:**

Risk Group 1 (High Risk Exclusive MSM)

$$\psi_{l,3,1}=(1-R_{l,3,1}) R_{l,3,2}$$

Risk Group 2 (High Risk Bisexual)

$$\psi_{l,3,2}={{(1-R}_{l,3,1})(1-R}_{l,3,2})$$

Risk Group 3 (Low Risk Bisexual)

$$\psi_{l,3,1}=R_{l,3,1}$$

where $R_{l,3,1}$ is the fraction of MSM who are low risk and bisexual, $R_{l,3,2}$ is the fraction of high risk MSM who are exclusively MSM (no female partners). This is based on data from the MARPs geographic mapping report [8]. Whether a risk group forms heterosexual partnerships in addition to homosexual partnerships has implications for the calculation of the force of infection (described further in Section 6).

HIV related mortality consists of both death from AIDS and death from AIDS following ART failure. The differential HIV related mortality between risk groups would mean that the proportion of the population in each risk group would vary over time without compensatory movements between risk groups.

The process used to hold the proportion of the population in each risk group over time constant is described below. It is turned on if the parameter ι=1 and off if ι=0, with the parameter ι included in the model fitting. The total number of heterosexual men (k=2) and women (k=1) in each risk group is calculated, and the value compared with the desired number in each risk group needed to match the defined risk structure. It is assumed there is no replacement in the MSM population or in the 50+ age group. $N_{k,l,r}$ is the total number of women (k=1) or heterosexual men (k=2) in each risk group, and these values are summed across risk groups to give the total number$T^{l,k}$ of women (k=1) or heterosexual men (k=2) in location l:

$$N_{k,l,r}=\sum_{s} \sum_{i} X_{r,i,1}^{s,l,k}$$

$$T^{l,k}=\sum_{r} N_{k,l,r}$$

The deficit within each group is calculated and individuals are moved between risk groups as needed according to the equations below:

**For both women and heterosexual men (k=1 and k=2 respectively):**

For the highest risk group (r=1):

$$G_{1}^{l,k}=\psi_{l,k,1}T^{l,k}-N_{k,l,1}$$

$${X_{1,i,1}^{s,l,k}\longrightarrow X_{1,i,1}^{s,l,k}+G}_{1}^{l,k}\frac{X_{2,i,1}^{s,l,k}}{N_{k,l,2}}$$

For the medium risk group (r=2):

$$G_{2}^{l,k}=\psi_{l,k,2}T^{l,k}-(N_{k,l,2}-G_{1}^{l,k})$$

$${X_{2,i,1}^{s,l,k}\longrightarrow X_{2,i,1}^{s,l,k}+G}_{2}^{l,k}\frac{X_{3,i,1}^{s,l,k}}{N_{k,l,3}}-G_{1}^{l,k}\frac{X_{2,i,1}^{s,l,k}}{N_{k,l,2}}$$

For the lowest risk group (r=3):

$$X_{3,i,1}^{s,l,k}\longrightarrow X_{3,i,1}^{s,l,k}-G_{2}^{l,k}\frac{X_{3,i,1}^{s,l,k}}{N_{k,l,3}}$$

Where $\psi_{l,k,r}$ is the target fraction in risk group r for sex k in location l and $G_{r}^{l,k}$ the calculated current deficit in number of individuals needed to match the target fraction in risk group r.

As previously described, whether these movements are used to hold the risk group sizes constant is one of the factors considered in the model fitting process for each location (further described in Section 7). Examination of the model fits suggests that movement between risk groups is turned on in the majority of locations (30 of 48). Removing these movements gives an epidemic profile which peaks earlier and does not reach as high prevalence levels, as the high infection rate in high risk groups leads to raised mortality, and so the size of the high risk group declines in the absence of movement into these populations. Additionally there is a steeper decline in prevalence following the peak when movements are removed, meaning that the balancing of risk groups is useful in capturing the plateauing of prevalence observed in the Kenyan prevalence data.

### The Incorporation of Interventions

A number of interventions are included in the model (Late ART, Circumcision, PrEP, Behaviour Change, and Accelerated ART). Accelerated ART is defined as ART initiated at any CD4 cell count via active outreach (i.e. individuals do not have to experience illness to seek treatment). Late ART assumes initiation at an average CD4 cell count of 200 cells per microliter, and corresponds to those actively seeking treatment due to ill health. Late ART is incorporated through the parameter $a^{l}\left( t \right)$which specifies the proportion of those in location l who will receive treatment. Accelerated ART and behaviour change are incorporated as a change in the rate of movement into the ART state and the partner change rate respectively. PrEP and circumcision are incorporated through the movement between intervention states (i). There are four intervention states in men:

Intervention group 1: no PrEP, not circumcised

Intervention group 2: no PrEP, circumcised

Intervention group 3: PrEP, not circumcised

Intervention group 4: PrEP, circumcised

There are two intervention states in women:

Intervention group 1: no PrEP

Intervention group 2: PrEP

*Existing Interventions*

Both behaviour change and ART scale up have taken place in Kenya. There is evidence that marked behaviour change has occurred in Kenya, with a decline in the proportion of the population who report multiple partnerships and an increase in reported condom use [11]. This behaviour change is incorporated as a change in the partner change rate and is specified as follows:

For t$\leq t_{BC}$

$$B_{1}^{l}=1$$

For $t_{BC}$< t <${(t}_{BC}+D_{BC})$

$$B_{1}^{l}=1+((t-t_{BC})(M_{l}-1))/D_{BC}$$

For t ≥${(t}_{BC}+D_{BC})$

$$B_{1}^{l}=M_{l}$$

where$M_{l}$ is the total relative change in partner change rate due to behaviour change, $t_{BC}$ the start time of behaviour change, and $D_{BC}$ the time before the specified relative change is reached. $B_{1}^{l}$ is used to adjust the partner change rates in Section 6 for calculation of the force of infection. The total relative change in partner change rate $M_{l}$and the start time of behaviour change $t_{BC}$are included in the fitting process Section 7.

Late ART is introduced at time $t_{ART}$ and is assumed to be scaled up at rate$a_{rate}^{l}$ until a defined proportion$a_{max}^{l}$ receive treatment. The rate of scale up of ART is informed by location specific estimates of coverage from the County Profiles Report [7] (see Section 8). It is assumed late ART is scaled up at an equal rate across all subgroups in that location. A ceiling of 90% coverage $(a_{max}^{l})$is assumed. Both the year when ART is introduced $t_{ART}$ and the rate of ART scale up $a_{rate}^{l}$ are included in the model fitting process (Section 7). The fraction of individuals who will receive late ART at time t is given by$a^{l}(t)$, defined as:

For t$\leq t_{ART}$

$$a^{l}(t)=0$$

For t$>t_{ART}$and $a^{l}\left( t \right)< a_{max}^{l}$

$$a^{l}(t)=a_{rate}^{l}(t-t_{ART})$$

For t $>t_{ART}$ and $a^{l}\left( t \right)= a_{max}^{l}$

$$a^{l}(t)=a_{max}^{l}$$

*Prospective Intervention Scenarios*

Four prospective interventions are considered as components of a combination prevention intervention to be implemented in each location. The interventions (behaviour change, PrEP, accelerated ART, and male circumcision) can be applied to low and medium risk women, Female Sex Workers (FSW), heterosexual men, and MSM as appropriate. Clients of sex workers are considered alongside lower risk men for intervention implementation. Key intervention related assumptions and the corresponding references used to inform them are given in Table S3 below.

Efficacy assumptions for male circumcision and accelerated ART interventions are based on observed efficacy in clinical trials [12]. The PrEP efficacy assumption is in line with similar modelling analyses informed by clinical trials [13].The efficacy of behaviour change interventions is based on an assumption that BCC can lead to modest reductions in risk behaviour. Whilst it has been difficult to demonstrate a sustained impact of behaviour change communication (BCC), it is likely that behaviour change has been a principle driver of reductions in HIV incidence over the last ten years in places such as Zimbabwe, Malawi and Uganda. Similar assumptions have been in the Investment Framework papers [14] and in many other analyses of prevention programming [15].

The target circumcision coverage reflects the current progress and aims of the VMMC program in Kenya [16]. Currently we assume that all of the population could be reached by a BCC campaign, with the analysis assuming 100% coverage of such an intervention as it is designed to reach the entire population.

The current values of each of these parameters is described in Table S3 are in line with assumptions made in other economic studies.

Table S3. The intervention related parameters.

| Intervention | Coverage Assumption | Efficacy Assumption | Unit Cost Value Used in the Analysis | Parameter Definition |
| --- | --- | --- | --- | --- |
| Male circumcision | Scaled up at a fixed rate: intervention unable to exceed 80% of eligible men, based on VMMC targets set [16]. | Risk of infection for circumcised man 60% less than for other men. [12, 17]. | $60 [14] | ϕ is the rate of scale up of circumcision |
| Behaviour Change Communication | The intervention is designed to reach 100% of the population- with the adjustment in the partner change rate applied to the mean partner change rate in the entirety of the risk group. | 20% reduction in risk for each low risk person reached, 50% reduction in risk for high risk groups (MSM and FSW)* | $20 in FSW and MSM, $10 in the low risk population [14] | $Z_{k,r}^{l}$ is the relative change in partner change rate due to the intervention. |
| Accelerated ART (in addition to late ART, representing outreach to those with initiation at higher CD4 counts) | Achievable coverage assumed to be 33% in low risk women and heterosexual men, 66% in FSW and MSM | 85% reduction in risk of transmission for a person on ART relative to others. [18] | $515 [13, 19, 20] | $A_{k,r}^{l}$ is the proportion of each group who receive accelerated ART |
| Pre-exposure prophylaxis | The maximum PrEP coverage is assumed to be 25% in low risk women and heterosexual men and 50% in FSW and MSM | 75% reduction in risk of infection for a person on PrEP relative to others. [13] | $250 [13] | $Y_{k,r}^{l}$ is the maximum PrEP coverage assumed in each group. |

* Here the coverage corresponds to the efficacy of the intervention; as such a programme would be provided to the entire population but will only alter the behaviour of a proportion of the population. The efficacy of the behaviour change intervention assumes that a modest change is possible in the general population, with a greater reduction in risk in the high risk populations. This is in line with the current opinion on behaviour change interventions as described in the investment framework papers and other analyses of prevention programming [14, 15, 21]. Although it is challenging to demonstrate sustained impact from behaviour change communication interventions; it is likely that such strategies were significant contributors to the decline in the epidemic in a number of countries, including Zimbabwe and Uganda [22].

Interventions are introduced at time $t_{int}$ (2015) and continue for a period of 15 years. The same efficacy assumptions are made in all locations (l).

As described in Section 3, the proportion of men entering the model who are circumcised is given by the parameter $F_{l}^{1}$ which describes the baseline proportion of men circumcised. During the intervention, circumcision is assumed to be scaled up at a fixed rate ϕ, however the implementation is capped such that if the proportion of men circumcised $F_{l}^{2}$(t) in location l at time t exceeds a threshold$F_{max}$, defined here as 80%, scale up ceases. This is based on the assumption that no intervention could circumcise greater than 80% of eligible men. To simulate the movement of men from uncircumcised to circumcised intervention groups, we calculate the proportion of men currently circumcised$F_{l}^{2}(t)$, and then adjust the rate of circumcision accordingly:

$$F_{l}^{2}(t) =\frac{\sum_{k=2}^{3} \sum_{s} \sum_{r} X_{r,2,1}^{s,l,k}+\sum_{k=2}^{3} \sum_{s} \sum_{r} X_{r,4,1}^{s,l,k}}{\sum_{k=2}^{3} \sum_{s} \sum_{r} \sum_{i} X_{r,i,1}^{s,l,k}}$$

For t ≤ $t_{int}$

$$n_{c}=0$$

For t>$t_{int}$and $F_{l}^{2}(t)$<$F_{max}$

$$n_{c}=\phi$$

For t>$t_{int}$and $F_{l}^{2} \left( t \right) \geq F_{max}$

$$n_{c}=0$$

where$n_{c}$ is the per capita rate of circumcision. The rate of circumcision is assumed to be the same in all men (across all risk groups in both heterosexual men and MSM), and is incorporated into the differential equations defining the movements between subgroups of HIV negative men.

The proportion of heterosexual men, MSM, FSW, and general population women who receive PrEP in each location is specified by the parameter $Y_{k,r}^{l}$ . The relevant sex and partnership type (denoted k) and risk groups (r) are used for the calculation of the required number of individuals moving between PrEP states (e.g. an intervention for FSW refers to (k=1) women and (r=1) for the highest risk group).

As described previously, there are four intervention groups in men (based on circumcision status and receipt of PrEP) and two in women (based on receipt of PrEP alone). Only HIV negative individuals receive PrEP. The movement between states $X_{r,i,a}^{s,l,k}$ due to a PrEP intervention is described as follows:

**For men (with four intervention groups (i)) for both heterosexual men (k=2) and MSM (k=3):**

$$O_{r}^{l,k}=X_{r,3,1}^{1,l,k}+X_{r,4,1}^{1,l,k}$$

$$S_{r}^{l,k}=\sum_{i}^{4} X_{r,i,1}^{1,l,k}$$

$$Q_{r}^{l,k}={Y_{k,r}^{l}S}_{r}^{l,k}-O_{r}^{l,k}$$

$$X_{r,1,1}^{1,l,k}\longrightarrow X_{r,1,1}^{1,l,k}-Q_{r}^{l,k}\frac{X_{r,1,1}^{1,l,k}}{{(S}_{r}^{l,k}-O_{r}^{l,k})}$$

$$X_{r,2,1}^{1,l,k}\longrightarrow X_{r,2,1}^{1,l,k}-Q_{r}^{l,k}\frac{X_{r,2,1}^{1,l,k}}{{(S_{r}^{l,k}-O}_{r}^{l,k})}$$

$$X_{r,3,1}^{1,l,k}\longrightarrow X_{r,3,1}^{1,l,k}+Q_{r}^{l,k}\frac{X_{r,1,1}^{1,l,k}}{{{(S}_{r}^{l,k}-O}_{r}^{l,k})}$$

$$X_{r,4,1}^{1,l,k}\longrightarrow X_{r,4,1}^{1,l,k}+Q_{r}^{l,k}\frac{X_{r,2,1}^{1,l,k}}{{(S}_{r}^{l,k}-O_{r}^{l,k})}$$

**For women (with two intervention groups (i) for both general population women (r=2 and r=3) and FSW (r=1)):**

$$O_{r}^{l,1}=X_{r,2,1}^{1,l,1}$$

$$S_{r}^{l,1}=\sum_{i}^{2} X_{r,i,1}^{1,l,1}$$

$$Q_{r}^{l,1}={Y_{1,r}^{l}S}_{r}^{l,1}-O_{r}^{l,1}$$

$$X_{r,1,1}^{1,l,1}\longrightarrow X_{r,1,1}^{1,l,1}-Q_{r}^{l,1}$$

$$X_{r,2,1}^{1,l,1}\longrightarrow X_{r,2,1}^{1,l,1}+Q_{r}^{l,1}$$

where$O_{r}^{l,k}$ is the total number of individuals in each group on PrEP (with the group defined by location l, sex and partnership preference k, and risk group r), $S_{r}^{l,1}$ the total number across all intervention states in each group, and $Q_{r}^{l,k}$ the deficit in the number on PrEP.

The proportion of each subpopulation who receives accelerated ART is described as$A_{k,r}^{l}$, defined for the relevant subpopulation (heterosexual men, MSM, FSW and general population women) which the intervention is targeted to. The rate of receiving accelerated ART, defined as $\omega_{r,s,l,k}$ , is dependent on the CD4 stage (s), sex and partner preference (k), risk group (r) and location (l).$\eta$ is the rate of flow onto Accelerated ART. Accelerated ART can be restricted to those in infection states s using the parameter $W_{s}$ which is 1 if Accelerated ART is available to state s and 0 otherwise.

For t$\leq t_{int}$:

$$\omega_{r,s,l,k}=0$$

For t >$t_{int}$:

$$\omega_{r,s,l,1}={\eta A}_{k,r}^{l}W_{s}$$

As with the behaviour change defined above, behaviour change implemented during the intervention is incorporated as a relative change$B_{2,r,i}^{l,k}$ to the baseline partner change rate. This magnitude of the relative change is defined as $Z_{k,r}^{l}$ for the relevant subpopulation (heterosexual men, MSM, FSW and general population women) which the intervention is targeted to.

For t${\leq t}_{int}$

$$B_{2,r,i}^{l,k}=1$$

For t >$t_{int}$

$$B_{2,r,i}^{l,1}=Z_{k,r}^{l}$$

### The Calculation of the Force of Infection

Each location is modelled independently, that is transmission occurs within counties or cities with no transmission considered between locations (l).

The force of infection $\lambda_{r,i}^{l,k}$ experienced by susceptible members of each subgroup is described below. The force of infection for a subgroup is the sum of the force of infection from all partnerships types with all other subgroups with which they are mixing. Both women (k=1) and heterosexual men (k=2) can form only heterosexual partnerships, MSM in risk group 1 (k=3, r=1) form homosexual partnerships only and MSM in risk group 2 and 3 (k=3, r=2, 3) form both homosexual and heterosexual partnerships. In the equations below, the risk group and intervention status of the group with which the group of interest is mixing are denoted with a prime ($r^{'}$for the risk group and $i^{'}$for the intervention status).

**For women (k=1) and heterosexual men (k=2) who can form heterosexual partnerships only:**

Women

$$\lambda_{r,i}^{l,1}=\sum_{r^{'}=1}^{3} \sum_{s^{'}=2}^{8} (c_{1,l,r,r^{'}}P_{1,l,r,r^{'}i,s^{'}})$$

Men

$$\lambda_{r,i}^{l,2}=\sum_{r^{'}=1}^{3} \sum_{s^{'}=2}^{8} (c_{2,l,r,r^{'}}P_{2,l,r,r^{'}i,s^{'}})$$

**For MSM (k=3):**

MSM

For r=1

$$\lambda_{1,i}^{l,3}=\sum_{r^{'}=1}^{3} \sum_{s^{'}=2}^{8} (c_{3,l,1,r^{'}}P_{3,l,1,r^{'}i,s^{'}} )$$

For r=2 and r=3

$$\lambda_{r,i}^{l,3}=\sum_{r^{'}=1}^{3} \sum_{s^{'}=2}^{8} (c_{2,l,r,r^{'}}P_{2,l,r,r^{'}i,s^{'}})+\sum_{r^{'}=1}^{3} \sum_{s^{'}=2}^{8} (c_{3,l,r,r^{'}}P_{3,l,r,r^{'}i,s^{'}} )$$

Where$c_{1,l,r,r^{'}}$ and $c_{2,l,r,r^{'}}$give the distribution of heterosexual and $c_{3,l,r,r^{'}}$homosexual partnerships across the subgroups of the population. The probability of transmission in a particular partnership is dependent on the partnership type, infection state of the partner, risk groups and interventions present in the subgroups mixing, and is defined as $P_{p,l,r,r^{'}i,s^{'}}$. The partnership type p is defined based on whether it is a heterosexual partnership formed by women (p=1), a heterosexual partnership formed by heterosexual men or MSM (p=2) or a homosexual partnership formed by MSM (p=3). The distribution of heterosexual partnerships formed by MSM of risk groups 2 and 3${(c}_{2,l,r,r^{'}})$ and the probability of transmission in a heterosexual partnership formed by MSM ($P_{2,l,r,r^{'}i,s^{'}}$) are both assumed to be identical to that formed by heterosexual men of the equivalent risk groups.

*Calculation of the Distribution of Heterosexual Partnerships*

The distribution of heterosexual partnerships is described below. When calculating the total number of men available to form heterosexual partnerships, MSM that have sex with women (MSMW) (in risk groups 2 and 3, defined by k=3, r=2, 3) are included in addition to the exclusively heterosexual men (k=2). They are assumed to have an identical heterosexual partner change rate as the equivalent risk groups in heterosexual men (r=2, r=3).

The partner change rate for each subpopulation is calculated from the mean number of heterosexual partnerships in men and women, the proportion of men and women in each risk group and the relative risk between risk groups.

For both women and men (k=1 and 2) the partner change rate in each risk group${( C}_{k,l,r}$) is calculated from the mean partner change rate for that sex ($\bar{c}_{l,k}$), the proportion of each sex k in risk group r ${(\psi}_{l,k,r}$ ) and the relative risk ($\varpi_{l,k,r}$) in relation to the lowest risk group (r=3), so that $(\varpi_{l,k,3}$=1). These parameters ($\bar{c}_{l,k}$,$R_{l,k,1}and R_{l,k,2}$used to calculate $\psi_{l,k,r}$ ,and$\varpi_{l,k,r}$) are all included in the model fitting process (Section 7).

$$C_{k,l,r}=\varpi_{l,k,r}\frac{\bar{c}_{l,k}}{\sum_{r} \varpi_{l,k,r}\psi_{l,k,r}}$$

The change rate is adjusted to incorporate the relative change due to both the background $B_{1}^{l}$and intervention $B_{2,r,i}^{l,k}$ mediated behaviour change (described previously in Section 5 the incorporation of interventions).

$${C_{k,l,r}^{*}=C_{k,l,r}B}_{1}^{l}B_{2,r,i}^{l,k}$$

Mixing between risk groups is controlled by the parameter ε, describing to what degree groups preferentially mix with others of their own risk group. ε is also included in the model fitting process (Section 7). If ε is equal to 1 mixing is fully assortative and if ε is equal to 0 mixing is random.$\delta_{r,r^{'}}$ is the Kronecker delta which is equal to one if r and r’ are equivalent and equal to zero otherwise. MSM (k=3) risk groups who are also able to form heterosexual partnerships are added to the total of heterosexual men according to the parameter$H_{r^{'}}$, which is 0 for risk group 1 and 1 otherwise. This reflects the fact that risk group one form homosexual partnerships exclusively but risk groups two and three can also form heterosexual partnerships. $n_{r^{'}}$is the number of risk groups defined for each sex ($n_{r^{'}}$=3) and $C_{k,l,r}^{*}$ is the adjusted partner change rate.

**For women:**

$$c_{1,l,r,r^{'}}=C_{1,l,r}^{*}\left( \varepsilon\delta_{r,r^{'}}+(1-\varepsilon)\frac{C_{2,l,r^{'}}^{*}{(N}_{2,l,r^{'}}+{H_{r^{'}}N}_{3,l,r^{'}})}{\sum_{u}^{n_{r^{'}}} {C_{2,l,u}^{*}(N}_{2,l,u}+{H_{u}N}_{3,l,u})} \right)$$

**For heterosexual men and MSMW:**

$$c_{2,l,r,r^{'}}=C_{2,l,r}^{*}\left( \varepsilon\delta_{r,r^{'}}+(1-\varepsilon)\frac{C_{1,l,r^{'}}^{*}N_{1,l,r^{'}}}{\sum_{u}^{n_{r^{'}}} C_{1,l,u}^{*}N_{1,l,u}} \right)$$

Where:

$$N_{k,l,r}=\sum_{s} \sum_{i} X_{r,i,1}^{s,l,k}$$

Partner change rates must be adjusted so that the number of partnerships formed between men and women balance. The imbalance$V_{l,r,r^{'}}$ is calculated for a pair of subgroups using the pattern of contacts ($c_{p,l,r,r^{'}}$) and number of individuals$N_{k,l,r}$ in each subgroup.

$$V_{l,r,r^{'}}=\frac{{((N}_{2,l,r^{'}}+{H_{r^{'}}N}_{3,l,r^{'}})c_{2,l,r^{'},r})}{N_{1,l,r}c_{1,l,r,r^{'}}}$$

The adjusted partner change rate is then calculated from the degree of imbalance V and the unadjusted partner change rate for each group. The adjusted partner change rate is specific to the partnership between the subgroups of interest.

$$c_{1,l,r,r^{'}}\to c_{1,l,r,r^{'}}{V_{l,r,r^{'}}}^{\theta}$$

$$c_{2,l,r^{'},r}\to{c_{2,l,r^{'},r}V_{l,r,r^{'}}}^{(\theta-1)}$$

The parameter θ describes the degree of compromise between the two sexes. It is set here to 0.5 meaning compromise is equal between the two groups.

*Calculation of the Distribution of Homosexual Partnerships*

The homosexual partner change rate in each risk group for MSM is defined as follows: $\varsigma_{H}$ is the defined partner change rate in high risk exclusive MSM, with the partner change rate in risk groups 2 and 3$(C_{3,l,2}$ and $C_{3,l,3})$ calculated from the male to female partner ratio for the high and low risk MSM group respectively ($\rho_{r}$) and the partner change rate for heterosexual men of the equivalent risk groups$C_{1,l,r}$.

$$C_{3,l,1}=\varsigma_{H}$$

$$C_{3,l,2}=\rho_{2}C_{1,l,2}$$

$$C_{3,l,3}=\rho_{3}C_{1,l,3}$$

The parameters$\varsigma_{H}$, $\rho_{2},$and $\rho_{3}$are all included in model fitting (Section 7). As with the heterosexual partnerships, the homosexual change rate can be adjusted due to a behaviour change intervention in this group, with the relative change given by $B_{2,r,i}^{l,3}$ :

$${C_{3,l,r}^{*}=C_{3,l,r}B}_{2,r,i}^{l,3}$$

Mixing in MSM is random and in proportion to the number of contacts offered by each risk group. The resulting partner change rate in MSM is as follows:

$$N_{3,l,r}=\sum_{s} \sum_{i} X_{r,i,1}^{s,l,3}$$

$$c_{3,l,r,r'}=\left( \frac{{C_{3,l,r}^{*}C_{3,l,r^{'}}^{*}N}_{3,l,r^{'}}}{\sum_{u}^{n_{r^{'}}} {C_{3,l,u}^{*}N}_{3,l,u}} \right)$$

No balancing is required for MSM as there are no constraints on the directionality of partnerships.

*The Probability of Transmission per Partnership*

Transmission is modelled per partnership, with the probability of becoming infected from a specific partnership $P_{p,l,r,r^{'},i{,s}^{'}}$ dependent on the prevalence of the infection state (s) in the group contacted and the probability of transmission. Different partnership types are assumed to be associated with a different probability of transmission, according to the partnership type, infection state, intervention status and risk groups of the groups mixing. The probability of transmission per partnership$P_{p,l,r,r^{'},i,s^{'}}$ is defined as:

**For heterosexual partnerships formed by women (p=1):**

$$P_{1,l,r,r^{'},i,s^{'}}=\frac{{\beta\kappa}_{1,r,r^{'}}\tau_{s^{'}}\xi_{1,i}\left( \sum_{i^{'}} X_{r^{'},i^{'},1}^{s^{'},l,2}+\sum_{i^{'}} {H_{r^{'}}X}_{r^{'},i^{'},1}^{s^{'},l,3} \right)}{\sum_{s^{'}=1}^{8} \left( \sum_{i^{'}} X_{r^{'},i^{'},1}^{s^{'},l,2}+\sum_{i^{'}} {H_{r^{'}}X}_{r^{'},i^{'},1}^{s^{'},l,3} \right)}$$

**For heterosexual partnerships formed by men (p=2):**

$$P_{2,l,r,r^{'},i,s^{'}}=\frac{{\beta\kappa}_{2,r,r^{'}}\tau_{s^{'}}\xi_{2,i}\sum_{i^{'}} X_{r^{'},i^{'},1}^{s^{'},l,1}}{\sum_{i^{'}} \sum_{s^{'}=1}^{8} X_{r^{'},i^{'},1}^{s^{'},l,1}}$$

**For homosexual partnerships formed by MSM (p=3):**

$$P_{3,l,r,r^{'},i,s^{'}}=\frac{{\beta\kappa}_{3,r,r^{'}}\tau_{s^{'}}\xi_{3,i}\sum_{i^{'}} X_{r^{'},i^{'},1}^{s^{'},l,3}}{\sum_{i^{'}} \sum_{s^{'}=1}^{8} X_{r^{'},i^{'},1}^{s^{'},l,3}}$$

where β is the baseline partnership transmission, $\kappa_{p,r,r^{'}}$the modifier factor based on the partnership type (p) and risk groups mixing, $\tau_{s^{'}}$the modifier factor based on infection state and $\xi_{p,i}$the modifier factor based on partnership type and intervention status. The baseline partnership transmission probability β is one of the parameters included in model fitting (Section 7). These modifier factors are defined in Table S4 - Table S6 below.

Partnerships between different risk groups will be associated with different probabilities of transmission, as such the modifier factor $\kappa_{p,r,r^{'}}$ is used to adjust the baseline transmission probability accordingly. For heterosexual partnerships (p=1 and p=2), depending on the risk groups mixing, a hierarchy is defined to determine the per partnership transmission probability as the risk group definitions are based on exclusions (e.g. commercial partnerships are only formed between men and women who are in the highest risk group). The lowest risk group has the baseline per partnership transmission probability by definition. This is adjusted to reflect the different per partnership transmission probabilities for both commercial and casual partnerships (Table S4). A similar set of rules govern the per partnership transmission probability for homosexual partnerships (p=3). All possible combinations of partnerships are described in Table S4 below, with high risk MSM those in risk groups 1 and 2 and low risk MSM in risk group 3. The baseline per partnership transmission probability is adjusted to reflect it is an MSM partnership. Where partnerships are between two high risk MSM groups a high risk MSM adjustment is made to the baseline transmission probability in addition to the MSM adjustment. Each of the parameters used to define the modifier factors ($\chi_{com}$,$\chi_{cas,} \chi_{MSM}$and $\chi_{HR}$) are included in model fitting (Section 7)

Table S4. The adjustment of the transmission probability according to the partnership type and risk classification of the groups mixing. The commercial, casual, MSM and high risk MSM adjustments are all parameters included in the model fitting process.

| Modifier Factor | Risk group | Risk Group of Partner | Definition |
| --- | --- | --- | --- |
| For heterosexual partnerships (p=1 and p=2): | | | |
| $\boldsymbol{\kappa}_{\boldsymbol{p,1,1}}$ | 1 | 1 | Commercial Adjustment$\chi_{com}$ |
| $\boldsymbol{\kappa}_{\boldsymbol{p,1,2}}$ | 1 | 2 | Casual Adjustment$\chi_{cas}$ |
| $\boldsymbol{\kappa}_{\boldsymbol{p,1,3}}$ | 1 | 3 | Baseline (no adjustment)  1 |
| $\boldsymbol{\kappa}_{\boldsymbol{p,2,1}}$ | 2 | 1 | Casual Adjustment$\chi_{cas}$ |
| $\boldsymbol{\kappa}_{\boldsymbol{p,2,2}}$ | 2 | 2 | Casual Adjustment  $\chi_{cas}$ |
| $\boldsymbol{\kappa}_{\boldsymbol{p,2,3}}$ | 2 | 3 | Baseline (no adjustment)  1 |
| $\boldsymbol{\kappa}_{\boldsymbol{p,3,1}}$ | 3 | 1 | Baseline (no adjustment)  1 |
| $\boldsymbol{\kappa}_{\boldsymbol{p,3,2}}$ | 3 | 2 | Baseline (no adjustment)  1 |
| $\boldsymbol{\kappa}_{\boldsymbol{p,3,3}}$ | 3 | 3 | Baseline (no adjustment)  1 |
| For Homosexual Partnerships | | | |
| $\boldsymbol{\kappa}_{\boldsymbol{3,1,1}}$  $\boldsymbol{\kappa}_{\boldsymbol{3,1,2}}$  $\boldsymbol{\kappa}_{\boldsymbol{3,2,1}}$  $\boldsymbol{\kappa}_{\boldsymbol{3,2,2}}$ | 1  1  2  2 | 1  2  1  2 | MSM High Risk Adjustment and MSM Adjustment  $\chi_{MSM}\chi_{HR}$ |
| $\boldsymbol{\kappa}_{\boldsymbol{3,1,3}}$  $\boldsymbol{\kappa}_{\boldsymbol{3,2,3}}$  $\boldsymbol{\kappa}_{\boldsymbol{3,3,1}}$  $\boldsymbol{\kappa}_{\boldsymbol{3,3,2}}$  $\boldsymbol{\kappa}_{\boldsymbol{3,3,3}}$ | 1  2  3  3  3 | 3  3  1  2  3 | MSM Adjustment  $\chi_{MSM}$ |

The modifier factor $\tau_{s^{'}}$ is used to define the adjustment based on infection stage (Table S5). This parameter is defined as a relative increase in transmissibility with respect to latent infection (>CD4 350 corresponding to infection states s=3 and s=4 in the model).

Table S5. The adjustment of the transmission probability by infection state.

| Modifier Factor | Infection State | Definition | Reference |
| --- | --- | --- | --- |
| $\boldsymbol{\tau}_{\boldsymbol{2}}$ | Acute | 27 | Hollingsworth et al 2008 [6] |
| $\boldsymbol{\tau}_{\boldsymbol{3}}$ | CD4>500 | 1 | By Definition |
| $\boldsymbol{\tau}_{\boldsymbol{4}}$ | CD4 350-500 | 1 | By Definition |
| $\boldsymbol{\tau}_{\boldsymbol{5}}$ | CD4 200-<350 | 6 | Donnell et al 2010 [23] |
| $\boldsymbol{\tau}_{\boldsymbol{6}}$ | CD4 <200 | 3.8 | Average of Hollingsworth et al 2008 [6] and Donnell et al 2010 [23] |
| $\boldsymbol{\tau}_{\boldsymbol{7}}$ | CD4<200 waiting for ART | 3.8 | Average of Hollingsworth et al 2008 [6] and Donnell et al 2010 [23] |
| $\boldsymbol{\tau}_{\boldsymbol{8}}$ | ART | 0.15 | Calculated as (1-ARTefficacy).  ART efficacy is assumed to be 85% [18]. |

Transmission is also modified in the presence of interventions by the modifier factor $\xi_{p,i}$ (Table S6). In men, this parameter is specific to whether the partnership is heterosexual or homosexual as circumcision is assumed to confer no protection against transmission between homosexual men.

Table S6. The adjustment of the transmission probability by intervention status.

| Modifier Factor | Intervention State | Definition | Reference |
| --- | --- | --- | --- |
| For heterosexual partnerships made by women (p=1): | | | |
| $\boldsymbol{\xi}_{\boldsymbol{1,1}}$ | No PrEP | 1 | By Definition |
| $\boldsymbol{\xi}_{\boldsymbol{1,2}}$ | PrEP | 0.25 | Assumes a 75% reduction in risk of acquisition of infection [13]. |
| For heterosexual partnerships made by men (p=2): | | | |
| $\boldsymbol{\xi}_{\boldsymbol{2,1}}$ | No PrEP, not circumcised | 1 | By Definition |
| $\boldsymbol{\xi}_{\boldsymbol{2,2}}$ | Circumcised, no PrEP | 0.4 | Assumed 60% reduction in acquisition of infection from women [24]. |
| $\boldsymbol{\xi}_{\boldsymbol{2,3}}$ | PrEP, not circumcised | 0.25 | Assumes a 75% reduction in risk of acquisition of infection [13]. |
| $\boldsymbol{\xi}_{\boldsymbol{2,4}}$ | PrEP and circumcised | 0.4*0.25 | Combined efficacy of circumcision and PrEP together. |
| For homosexual partnerships (p=3): | | | |
| $\boldsymbol{\xi}_{\boldsymbol{3,1}}$ | No PrEP, not circumcised | 1 | By Definition |
| $\boldsymbol{\xi}_{\boldsymbol{3,2}}$ | Circumcised, no PrEP | 1 | It is assumed that circumcision provides no protection from transmission between MSM. |
| $\boldsymbol{\xi}_{\boldsymbol{3,3}}$ | PrEP, not circumcised | 0.25 | Assumes a 75% reduction in risk of acquisition of infection [13]. |
| $\boldsymbol{\xi}_{\boldsymbol{3,4}}$ | PrEP and circumcised | 0.25 | Assumes a 75% reduction in risk of acquisition of infection due to PrEP and no protection due to circumcision from transmission between MSM. |

### Model Fitting

The model fitting process involves the optimisation of parameters to best match the prevalence (informed by Table S8 - Table S13), proportion of men circumcised (informed by Table S20 and Table S21) and proportion of individuals who receive ART (informed by Table S20 and Table S21) observed in each location (counties/cities) in Kenya.

For each parameter predefined bounds were specified based on the range of plausible values from existing information. The parameter bounds are described in Table S7 below. For the probabilities of HIV transmission, the defined ranges were directly informed by the literature [6, 23]. Bounds for sexual behaviour parameters were more vaguely defined in accordance with common understanding on the difficulties of direct measurement of sexual behaviour and pattern of sexual partnership formation.

Through drawing values from the defined bounds for each input parameter a full test parameter set is produced. How well the model realisation under this parameter set matches available data can be assessed through calculation of the likelihood. When assessing the fit, we compare the model realisation with a number of sources of observed data for each modelled location; including information on the level of ART coverage in the population, the level of circumcision in men and estimates of prevalence for men, women and MSM.

The likelihood function describes the probability of the observed data given the model and the parameter set. The form of the likelihood function reflects the probability distribution which best describes the process modelled. Here, we examine the proportion of a population with a given attribute (i.e. proportion HIV positive, on ART or circumcised) and as such the binomial model is used. The likelihood function for the binomial model is of the general form:

$$L(p|n,x)=\left( \begin{aligned} n \\ x \end{aligned} \right)p^{x}{(1-p)}^{n-x}$$

where n is the sample size of the observed data, x the proportion of individuals in the observed data with the attribute of interest, and p is the proportion of the modelled population with the attribute of interest. In this way, $L(p|n,x)$ describes the likelihood of p, given the data n and x, and so allows us to assess how well the model realisation fits to the observed data.

As we wish to evaluate the fit of the model to a number of different types of data, we need to produce a likelihood function which combines all of these different attributes of interest. The likelihood which we use to asses model fit is the product of a number of components; $L_{1}$ the ANC prevalence data, $L_{2}$ and $L_{3}$the DHS prevalence data for men and women respectively, $L_{4}$ the estimated coverage of ART in the population, $L_{5}$ circumcision in men, $L_{6}$ the prevalence in low risk MSM and $L_{7}$ the prevalence in high risk MSM.

The data used to assess each of these features are taken from a number of sources, and is described in the Input Tables given in Section 8. The prevalence levels for men and women were taken from the 2008 DHS survey, with the prevalence by county estimated using an interpolated map of prevalence using the DHS cluster data. It was adjusted to give estimates for men and women using the observed province level sex prevalence ratio from the DHS data. Similarly the level of ART coverage in the model and the level of circumcision in men in the model were compared with county level data from 201 The prevalence levels for low risk and high risk MSM was also compared with available data. It is assumed that low risk MSM have approximately double the prevalence of that in the heterosexual male population, and prevalence estimates in high risk MSM in cities are taken from earlier studies.

All of the components of the likelihood function are calculated using the general equation described above with the relevant data sources, with the exception of $L_{1}$ the ANC prevalence data which combines data from spanning over 14 years. To give $L_{1}$ the ANC component of the likelihood, the likelihood of the model given the ANC data was evaluated for each year y and the product of these used.

$$L_{1}=\prod_{1}^{y} {p_{y}^{x_{y}}(1-p_{y})}^{n_{y}-x_{y}}$$

The likelihood of the observed data given the model realisation is then the product of components$L_{1}$ to $L_{7}$:

$$L_{tot}=\prod_{1}^{7} L_{n}$$

The parameter set which maximises the likelihood function represents the optimal ‘fit’ of the model. When implementing this calculation however, we calculate the log likelihood rather than the likelihood as this is computationally more favourable, and find the parameter set which minimises the negative log likelihood (which is equivalent to maximising the likelihood). To search the parameter space, the Nelder-Mead algorithm is used in order to find the input parameter set which gives the best fit to observed data (maximise the likelihood) for each location. As described above, parameters are drawn from the predefined bounds and for each resulting model realisation the likelihood is calculated from key model outputs and the corresponding observed data. The algorithm searches the parameter space and identifies the parameter set with the maximum likelihood and as such gives the best fit to the data. This algorithm is seeded 50 times using a set of parameters drawn from a latin hypercube sample to avoid confining the algorithm to local optima.

Table S7. The parameter bounds used in the model fitting process.

| Parameter | Explanation | Range |
| --- | --- | --- |
| $\boldsymbol{T}_{\boldsymbol{0}}$ | The start time of the epidemic | 1980-1990 |
| $\boldsymbol{\varepsilon}$ | Used in the calculation of the pattern of contacts between risk groups | 0.1-0.9 |
| ι | Whether replacement of high risk groups is turned on. If ι=0 replacement is off. If ι=1 replacement is on. Further details of the replacement of high risk groups are given in Section 4 | 0 or 1 |
| ${\bar{\boldsymbol{c}}}_{\boldsymbol{l,2}}$ | Mean partner change rate in men (per year) | 1-3 |
| ${\bar{\boldsymbol{c}}}_{\boldsymbol{l,1}}$ | Mean partner change rate in women (per year) | 1-3 |
| $\boldsymbol{R}_{\boldsymbol{l,2,2}}$ | Fraction of the male population who have casual sex | 0.1-0.6 |
| $\boldsymbol{R}_{\boldsymbol{l,2,1}}$ | Fraction of those men who have casual sex who are clients of FSW | 0.1-0.6 |
| $\boldsymbol{R}_{\boldsymbol{l,1,2}}$ | Fraction of the female population who have casual sex | 0.1-0.6 |
| $\boldsymbol{R}_{\boldsymbol{l,1,1}}$ | Fraction of those women who have casual sex who are FSW | 0.1-0.6 |
| $\boldsymbol{\varpi}_{\boldsymbol{l,2,2}}$ | The relative risk of risk group 2 compared to risk group 3in men- used to calculate the partner change rate in risk group 2 | 2-20 |
| $\boldsymbol{\varpi}_{\boldsymbol{l,2,1}}$ | The relative risk of risk group 1 compared to risk group 3 in men- used to calculate the partner change rate in risk group 1 | 20-50 |
| $\boldsymbol{\varpi}_{\boldsymbol{l,1,2}}$ | The relative risk of risk group 2 compared to risk group 3 in women- used to calculate the partner change rate in risk group 2 | 2-20 |
| $\boldsymbol{\varpi}_{\boldsymbol{l,1,1}}$ | The relative risk of risk group 1 compared to risk group 3 in women- used to calculate the partner change rate in risk group 1 | 20-50 |
| β | The baseline partnership transmission probability | 0.02-0.20 |
| $\boldsymbol{\chi}_{\boldsymbol{MSM}}$ | The transmission probability modifier factor for MSM | 5-4 |
| $\boldsymbol{\chi}_{\boldsymbol{com}}$ | The transmission probability modifier factor for commercial partnerships | 0.01-1 |
| $\boldsymbol{\chi}_{\boldsymbol{cas}}$ | The transmission probability modifier factor for casual partnerships | 0.05-0.5 |
| $\boldsymbol{\chi}_{\boldsymbol{HR}}$ | The transmission probability modifier factor for high risk MSM | 0.01-0.5 |
| $\boldsymbol{\rho}_{\boldsymbol{3}}$ | The male to female partner ratio in low risk MSM | 1-3 |
| $\boldsymbol{\rho}_{\boldsymbol{2}}$ | The male to female partner ratio in high risk MSM | 1-10 |
| $\boldsymbol{\varsigma}_{\boldsymbol{H}}$ | The partner change rate in high risk MSM | 1-50 |
| $\boldsymbol{F}_{\boldsymbol{l}}^{\boldsymbol{1}}$ | The baseline proportion of men circumcised | Defined such that it is 0.95-05 of the input proportion of men circumcised taken from the county profiles [7]. |
| $\boldsymbol{a}_{\boldsymbol{rate}}^{\boldsymbol{l}}$ | ART scale up rate | Defined such that it is 0.33-3 of one sixth of the ART coverage level taken from the county profiles [7]. |
| $\boldsymbol{t}_{\boldsymbol{ART}}$ | ART scale up year | 2003-2007 |
| $\boldsymbol{t}_{\boldsymbol{BC}}$ | Behaviour change start year | 1995-2010 |
| $\boldsymbol{M}_{\boldsymbol{l}}$ | Background behaviour change- relative change in mean partner change rate | 0.2-0 |

### Input Parameter Tables

The input data are described in a number of constituent tables (Table S8 - Table S21 below).

Table S8 and Table S9 describe the estimated prevalence in each county from the 2008/2009 DHS map (the methods used to produce this map are described in Section 9), and Table S10 and

Table S11 the estimated prevalence in each county from the ANC maps (also described further in Section 9) [25]. Table S12 and Table S13 gives the assumed prevalence ratio between the sexes, based on a province level analysis of the DHS 2008/2009 data [10]. Counties were allocated the appropriate province level estimate based on province membership. For the prevalence ratio in cities, estimates came from an analysis of the urban areas of each province from the DHS 2008/2009 [10].

Table S8. Estimated HIV prevalence by county (taken from the DHS 2008/2009 Map).

| County | Estimated Prevalence | County | Estimated Prevalence |
| --- | --- | --- | --- |
| Baringo | 2 | **Marsabit** | 5 |
| Bomet | 5.2 | **Meru** | 5.4 |
| Bungoma | 4.9 | **Migori** | 19.2 |
| Busia | 17 | **Murang'a** | 4.7 |
| Embu | 3.0 | **Nakuru** | 5.6 |
| Garissa | 0.3 | **Nandi** | 3.3 |
| Homa Bay | 21 | **Narok** | 8.4 |
| Isiolo | 1 | **Nyamira** | 5.9 |
| Kajiado | 5.7 | **Nyandarua** | 4.7 |
| Kakamega | 6.4 | **Nyeri** | 4.3 |
| Elgeyo-Marakwet | 4.3 | **Samburu** | 0 |
| Kericho | 8.0 | **Siaya** | 18.5 |
| Kiambu | 4.9 | **Taita Taveta** | 4.8 |
| Kilifi | 6.0 | **Tana River** | 2 |
| Kirinyaga | 4.2 | **Tharaka** | 4.3 |
| Kisii | 7.8 | **Trans Nzoia** | 7.8 |
| Kisumu | 15 | **Turkana** | 0.9 |
| Kitui | 3.4 | **Uasin Gishu** | 3.8 |
| Kwale | 3.3 | **Vihiga** | 8.6 |
| Laikipia | 5 | **Wajir** | 4 |
| Lamu | 0.4 | **West Pokot** | 4 |
| Machakos | 8 |  | |
| Makueni | 4.7 |  |  |
| Mandera | 7 |  |  |

Table S9. Estimated HIV prevalence for each city (taken from the DHS 2008/2009 Map).

| City | Estimated Prevalence |
| --- | --- |
| Nairobi | 7.2 |
| Mombasa | 3.3 |
| Kisumu | 15 |

Table S10. Time trend in HIV prevalence by county (taken from the ANC data based maps).

| County | Year | | | | | | | | | | | | | |
| --- | --- | --- | --- | --- | --- | --- | --- | --- | --- | --- | --- | --- | --- | --- |
|  | **2011** | **2010** | **2008** | **2006** | **2005** | **2004** | **2003** | **2002** | **2001** | **1998** | **1997** | **1995** | **1993** | **1990** |
| Baringo | 5.3 | 4.8 | 5.6 | 5.8 | 5.0 | 6.1 | 7.2 | 8.4 | 10.1 | 13 | 13.0 | 15.4 | 15 | 6.5 |
| Bomet | 10.7 | 7.7 | 7.0 | 5.9 | 6.8 | 7.1 | 10.3 | 11 | 13.3 | 14.4 | 14.9 | 13.1 | 10.5 | 6.4 |
| Bungoma | 5.0 | 4.5 | 5.5 | 5.5 | 6.1 | 6.1 | 9.2 | 8.6 | 15.5 | 19 | 17 | 13 | 10.7 | 7.3 |
| Busia | 8.6 | 7.3 | 6.3 | 7.0 | 9.6 | 10.0 | 14.1 | 10 | 13.6 | 19.6 | 18.8 | 16.4 | 16.0 | 15 |
| Embu | 5.7 | 4.7 | 5.3 | 4.4 | 4.3 | 4.8 | 7.0 | 5.5 | 8.3 | 15 | 19.5 | 10.0 | 10.2 | 5 |
| Garissa | 3.5 | 3.4 | 4.4 | 3.1 | 3.2 | 9 | 4.5 | 5.3 | 9.4 | 9.2 | 9.9 | 8.2 | 6.5 | 3.6 |
| Homa Bay | 20.4 | 15.0 | 17 | 13.7 | 17.3 | 16.7 | 24.0 | 24 | 29 | 17.8 | 16.9 | 12 | 9.7 | 8.7 |
| Isiolo | 4.9 | 3.6 | 3.5 | 5.0 | 4.5 | 5.3 | 8.5 | 7.2 | 10.6 | 17 | 14 | 10.6 | 7.4 | 3.5 |
| Kajiado | 5.9 | 5.9 | 5.6 | 5.4 | 4.8 | 5.7 | 6.8 | 7.1 | 14 | 16 | 10.7 | 10.1 | 14.2 | 4.0 |
| Kakamega | 7.4 | 6.9 | 7.8 | 6.8 | 7.9 | 8.3 | 16 | 12 | 13.2 | 14.7 | 19 | 17 | 10.2 | 6.6 |
| Elgeyo-Marakwet | 5.7 | 5.2 | 6.3 | 5.3 | 5.3 | 5.9 | 7.7 | 9.0 | 10.5 | 10.7 | 10 | 13.0 | 10.4 | 6.0 |
| Kericho | 7.8 | 6.4 | 6.3 | 5.9 | 5.9 | 6.3 | 7.8 | 8.9 | 10.2 | 12 | 18 | 14.5 | 13.1 | 8.4 |
| Kiambu | 7.5 | 5.8 | 5.7 | 6.1 | 7.1 | 8.4 | 8.8 | 8.3 | 11 | 16.7 | 12 | 15.5 | 19.6 | 3.5 |
| Kilifi | 4.3 | 3.5 | 4.1 | 4.2 | 4.2 | 4.0 | 5.4 | 6.3 | 9.5 | 17.3 | 13.4 | 15.5 | 13.8 | 7.2 |
| Kirinyaga | 6.5 | 4.7 | 5.3 | 4.7 | 5.1 | 5.8 | 7.0 | 6.9 | 9.4 | 18 | 13.7 | 13.2 | 9.4 | 6 |
| Kisii | 13.3 | 6.9 | 5.9 | 3.1 | 6.1 | 5.8 | 10.1 | 10.3 | 14.5 | 14.0 | 15.3 | 5.7 | 3.8 | 3 |
| Kisumu | 14.0 | 17 | 11 | 9.6 | 9.4 | 9.6 | 15.8 | 16.5 | 18.3 | 20.9 | 20.4 | 17.5 | 15.0 | 13.2 |
| Kitui | 5.1 | 5.1 | 4.9 | 5.6 | 4.7 | 5.0 | 5.4 | 5.8 | 9.2 | 14 | 10.7 | 7.9 | 9.3 | 3 |
| Kwale | 7.8 | 5.7 | 6.7 | 6.5 | 6.8 | 7.0 | 9.1 | 8.1 | 9.9 | 24 | 14.5 | 18.3 | 15.1 | 8.1 |
| Laikipia | 5.2 | 4.3 | 4.5 | 5.6 | 4.8 | 6.1 | 8.1 | 7.7 | 10.2 | 13.9 | 18 | 15.5 | 9.9 | 4.4 |
| Lamu | NA | 4.2 | 4.7 | 4.5 | 4.2 | 4.3 | 5.6 | 6.0 | 9.4 | 13.5 | 13 | NA | 10.4 | NA |
| Machakos | 6.6 | 5.7 | 6.4 | 5.5 | 5.9 | 6.2 | 6.3 | 7.3 | 18 | 14.9 | 15 | 10.8 | 15.7 | 6 |
| Makueni | 5.4 | 5.9 | 5.5 | 5.3 | 4.5 | 5.1 | 5.3 | 6.2 | 9.2 | 16 | 10.2 | 8.2 | 12 | 9 |
| Mandera | 5.8 | 6 | 6 | 4.6 | 3.6 | 4.2 | 6.2 | 7.0 | 8.1 | 14 | 18 | NA | 8.8 | NA |
| Marsabit | 7.3 | 8 | 6 | 6.1 | 5.0 | 6.9 | 9.3 | 9.7 | 10.5 | 10.6 | 10.9 | 18 | 8.9 | 5.6 |
| Meru | 5.1 | 3.6 | 7 | 4.7 | 4.9 | 4.5 | 8.1 | 6.0 | 10.1 | 17.0 | 13.6 | 9.6 | 3.7 | 4 |
| Migori | 17.4 | 10.5 | 7.1 | 7.9 | 9.4 | 10.7 | 15.7 | 15.2 | 17.6 | 16.0 | 15.8 | 10.0 | 8.1 | 7.3 |
| Murang'a | 7.0 | 4.5 | 5.6 | 4.8 | 6.0 | 7.3 | 7.2 | 7.5 | 9.4 | 12 | 10 | 15.6 | 17.0 | 6 |
| Nakuru | 6.3 | 5.5 | 5.5 | 7.1 | 6.1 | 7.0 | 8.1 | 8.9 | 10.5 | 15.3 | 15.8 | 20.2 | 17.6 | 7.1 |
| Nandi | 6.2 | 5.4 | 5.7 | 5.4 | 5.3 | 5.8 | 6.5 | 7.9 | 9.1 | 9.4 | 10.8 | 13.5 | 13 | 8.8 |
| Narok | 9.3 | 6.6 | 5.5 | 5.9 | 6.3 | 7.4 | 9.7 | 9.9 | 13.1 | 14.4 | 14.0 | 14.0 | 13 | 5.6 |
| Nyamira | 14 | 8.4 | 7.3 | 4.8 | 7.4 | 7.1 | 10 | 14 | 15.4 | 14.7 | 15.6 | 8.1 | 5.6 | 3.9 |
| Nyandarua | 5.9 | 4.6 | 5.0 | 5.9 | 5.7 | 7.3 | 7.8 | 7.5 | 9.4 | 16 | 15 | 19.6 | 14.4 | 5.5 |
| Nyeri | 5.9 | 4.5 | 4.6 | 4.9 | 5.5 | 6.2 | 7.9 | 7.6 | 10.2 | 13.2 | 9.0 | 17.9 | 5.6 | 6 |
| Samburu | 5.3 | 4.3 | 8 | 6.3 | 4.4 | 7.0 | 16 | 10.5 | 16 | 15 | 14 | 13.2 | 9.3 | 4.7 |
| Siaya | 15.1 | 16 | 18 | 10.7 | 13.1 | 13.3 | 19.1 | 17.6 | 18.9 | 20.2 | 17.7 | 15.5 | 13.8 | 15 |
| Taita Taveta | 5.7 | 8.2 | 5.4 | 5.7 | 3.7 | 4.2 | 4.7 | 5.7 | 7.4 | 16.7 | 15 | 13.0 | 13.0 | 5.6 |
| Tana River | 3.9 | 4.2 | 4.6 | 4.0 | 3.6 | 3.5 | 4.6 | 5.5 | 9.1 | 12 | 10.6 | 9.1 | 8.5 | 3.8 |
| Tharaka | 5.3 | 4.1 | 3.9 | 4.4 | 4.4 | 4.4 | 7.5 | 5.5 | 8.8 | 13.9 | 17.4 | 9.5 | 5.2 | 4 |
| Trans Nzoia | 5.0 | 6.0 | 8.0 | 5.1 | 5.6 | 6.4 | 9.7 | 12 | 13.9 | 9.4 | 19 | 10.1 | 8.1 | 3.9 |
| Turkana | 8.2 | 4.2 | 4.3 | 7.0 | 6.2 | 7.7 | 8.8 | 10.3 | 10.4 | 8.7 | 9.6 | 14 | 9.0 | 9.3 |
| Uasin Gishu | 5.7 | 5.3 | 6.0 | 5.2 | 5.2 | 5.9 | 7.2 | 8.7 | 10.0 | 9.6 | 10 | 18 | 10.7 | 6.4 |
| Vihiga | 14 | 10.7 | 10.3 | 7.4 | 9.3 | 10.3 | 19 | 15.0 | 15.7 | 17.9 | 16.4 | 14.2 | 13.6 | 12 |
| Wajir | 5.1 | 8 | 3.0 | 4.7 | 4.0 | 4.3 | 6.7 | 6.4 | 9.5 | 13 | 13 | 10.8 | 8.2 | 4.2 |
| West Pokot | 5.8 | 5.2 | 6.4 | 5.6 | 5.8 | 6.0 | 9.1 | 8.9 | 10.3 | 9.8 | 10.6 | 16 | 8.7 | 7.0 |

Table S11. Time trend in HIV prevalence by city (taken from the ANC data based maps).

| City | Year | | | | | | | | | | | | | |
| --- | --- | --- | --- | --- | --- | --- | --- | --- | --- | --- | --- | --- | --- | --- |
|  | **2011** | **2010** | **2008** | **2006** | **2005** | **2004** | **2003** | **2002** | **2001** | **1998** | **1997** | **1995** | **1993** | **1990** |
| Nairobi | 8.5 | 7.1 | 6.9 | 8.3 | 8.4 | 9.2 | 10.0 | 10.5 | 15.6 | 18.3 | 13.4 | 15.7 | 17.8 | 4.6 |
| Mombasa | 10.6 | 5.8 | 8.0 | 5.9 | 10.4 | 9.6 | 14.8 | 13.5 | 13.4 | 16.0 | 16.0 | 16.1 | 16.0 | 9.0 |
| Kisumu | 14.0 | 17 | 11 | 9.6 | 9.4 | 9.6 | 15.8 | 16.5 | 18.3 | 20.9 | 20.4 | 17.5 | 15.0 | 13.2 |

Table S12. Prevalence sex ratio by county (taken from analysis of the DHS 2008/2009 data).

| County | Prevalence Sex Ratio W:M | County | Prevalence Sex Ratio  W:M |
| --- | --- | --- | --- |
| Baringo | 7 | **Marsabit** | 3 |
| Bomet | 7 | **Meru** | 3 |
| Bungoma | 8 | **Migori** | 4 |
| Busia | 8 | **Murang'a** | 6 |
| Embu | 3 | **Nakuru** | 7 |
| Garissa | 1 | **Nandi** | 7 |
| Homa Bay | 4 | **Narok** | 7 |
| Isiolo | 3 | **Nyamira** | 4 |
| Kajiado | 7 | **Nyandarua** | 6 |
| Kakamega | 8 | **Nyeri** | 6 |
| Elgeyo-Marakwet | 7 | **Samburu** | 7 |
| Kericho | 7 | **Siaya** | 4 |
| Kiambu | 6 | **Taita Taveta** | 2 |
| Kilifi | 2 | **Tana River** | 2 |
| Kirinyaga | 6 | **Tharaka** | 3 |
| Kisii | 4 | **Trans Nzoia** | 7 |
| Kisumu | 4 | **Turkana** | 7 |
| Kitui | 3 | **Uasin Gishu** | 7 |
| Kwale | 2 | **Vihiga** | 8 |
| Laikipia | 7 | **Wajir** | 1 |
| Lamu | 2 | **West Pokot** | 7 |
| Machakos | 3 |  | |
| Makueni | 3 |  |  |
| Mandera | 1 |  |  |

Table S13. Prevalence sex ratio by city (taken from analysis of the DHS 2008/2009 data).

| City | Prevalence Sex Ratio  W:M |
| --- | --- |
| Nairobi | 7 |
| Mombasa | 3.0 |
| Kisumu | 8 |

Table S14 and Table S15 describe the demographic characteristics of each county and city respectively. In Table S14, the total population size is taken from the County Factsheets [7] and the age structure is taken from the 2009 census available from Kenya Open Data [26]. In Table S15, the data is informed by the County Service Delivery Profiles and the population size of urban/peri-urban areas reported in the MARPs geographic mapping report and from the 2009 census data [7, 8, 26].

Table S14. The demographic characteristics of each county.

| County | Total Population Size (Estimated 2011) | Proportion of the Male Population 15-49 | Proportion of the Male Population 50+ | Proportion of the Female Population 15-49 | Proportion of the Female Population 50+ |
| --- | --- | --- | --- | --- | --- |
| Baringo | 588478 | 0.42 | 0.08 | 0.44 | 0.09 |
| Bomet | 766861 | 0.45 | 0.08 | 0.46 | 0.08 |
| Bungoma | 1694695 | 0.42 | 0.08 | 0.44 | 0.09 |
| Busia | 507139 | 0.41 | 0.09 | 0.44 | 0.10 |
| Embu | 532852 | 0.50 | 0.12 | 0.50 | 0.14 |
| Garissa | 394804 | 0.43 | 0.08 | 0.46 | 0.06 |
| Homa Bay | 1006756 | 0.41 | 0.09 | 0.44 | 0.10 |
| Isiolo | 147928 | 0.45 | 0.10 | 0.46 | 0.10 |
| Kajiado | 727849 | 0.51 | 0.07 | 0.52 | 0.06 |
| Kakamega | 1725211 | 0.42 | 0.09 | 0.45 | 0.10 |
| Elgeyo-Marakwet | 391900 | 0.43 | 0.09 | 0.44 | 0.10 |
| Kericho | 803025 | 0.48 | 0.08 | 0.48 | 0.08 |
| Kiambu | 1692651 | 0.55 | 0.10 | 0.55 | 0.10 |
| Kilifi | 1175021 | 0.42 | 0.09 | 0.45 | 0.10 |
| Kirinyaga | 550745 | 0.54 | 0.12 | 0.53 | 0.14 |
| Kisii | 1203648 | 0.43 | 0.09 | 0.47 | 0.10 |
| Kisumu | 623791 | 0.47 | 0.08 | 0.48 | 0.10 |
| Kitui | 1045268 | 0.39 | 0.11 | 0.44 | 0.13 |
| Kwale | 688398 | 0.42 | 0.09 | 0.45 | 0.09 |
| Laikipia | 422718 | 0.47 | 0.10 | 0.48 | 0.11 |
| Lamu | 107546 | 0.49 | 0.10 | 0.47 | 0.10 |
| Machakos | 1134005 | 0.48 | 0.12 | 0.48 | 0.13 |
| Makueni | 913015 | 0.43 | 0.11 | 0.44 | 0.14 |
| Mandera | 649970 | 0.40 | 0.07 | 0.41 | 0.05 |
| Marsabit | 300589 | 0.43 | 0.10 | 0.43 | 0.10 |
| Meru | 1400032 | 0.48 | 0.12 | 0.49 | 0.12 |
| Migori | 958055 | 0.42 | 0.07 | 0.44 | 0.08 |
| Murang'a | 982921 | 0.46 | 0.15 | 0.47 | 0.17 |
| Nakuru | 1697951 | 0.50 | 0.08 | 0.50 | 0.08 |
| Nandi | 797427 | 0.46 | 0.09 | 0.46 | 0.09 |
| Narok | 900860 | 0.42 | 0.07 | 0.44 | 0.07 |
| Nyamira | 624921 | 0.44 | 0.10 | 0.48 | 0.09 |
| Nyandarua | 625350 | 0.45 | 0.10 | 0.47 | 0.11 |
| Nyeri | 723392 | 0.51 | 0.14 | 0.51 | 0.16 |
| Samburu | 237212 | 0.41 | 0.07 | 0.42 | 0.08 |
| Siaya | 879853 | 0.41 | 0.11 | 0.43 | 0.15 |
| Taita Taveta | 301516 | 0.50 | 0.12 | 0.48 | 0.14 |
| Tana River | 254348 | 0.39 | 0.09 | 0.43 | 0.08 |
| Tharaka | 377099 | 0.44 | 0.10 | 0.47 | 0.11 |
| Trans Nzoia | 867219 | 0.44 | 0.08 | 0.45 | 0.08 |
| Turkana | 905842 | 0.46 | 0.07 | 0.47 | 0.08 |
| Uasin Gishu | 946757 | 0.51 | 0.08 | 0.51 | 0.08 |
| Vihiga | 576283 | 0.39 | 0.14 | 0.43 | 0.15 |
| Wajir | 419438 | 0.40 | 0.08 | 0.42 | 0.06 |
| West Pokot | 543054 | 0.40 | 0.07 | 0.42 | 0.07 |

Table S15. The demographic characteristics of each city.

| City | Population Size (urban and peri-urban areas from the MARPs report) | Proportion of the Male Population 15-49 | Proportion of the Male Population 50+ | Proportion of the Female Population 15-49 | Proportion of the Female Population 50+ |
| --- | --- | --- | --- | --- | --- |
| Nairobi | 3,351,315 | 0.64 | 0.06 | 0.64 | 0.04 |
| Mombasa | 994,575 | 0.61 | 0.07 | 0.60 | 0.06 |
| Kisumu | 388,311 | 0.53 | 0.07 | 0.52 | 0.07 |

Table S16 and Table S17 describe the risk behaviour by county and city respectively, informed from the 2008/2009 DHS data [10]. In Table S16 , the analysis was conducted at province level, with constituent counties assigned the province level estimates. In Table S17, the analysis was restricted to the urban areas of the province which the city is located in.

Table S16. Risk behaviour by county taken from the DHS 2008/2009.

| County | Proportion of Men Risk Group 1 (Reporting Commercial Sex) | Proportion of Men Risk Group 2 (Reporting Casual Sex) | Proportion of Women Risk Group 1 (Reporting Commercial Sex) | Proportion of Women Risk Group 2 (Reporting Casual Sex) |
| --- | --- | --- | --- | --- |
| Baringo | 0.01 | 0.20 | 0.02 | 0.12 |
| Bomet | 0.01 | 0.20 | 0.02 | 0.12 |
| Bungoma | 0.03 | 0.24 | 0.02 | 0.08 |
| Busia | 0.03 | 0.24 | 0.02 | 0.08 |
| Embu | 0.01 | 0.18 | 0.03 | 0.08 |
| Garissa | 0.01 | 0.02 | 0.00 | 0.01 |
| Homa Bay | 0.01 | 0.26 | 0.03 | 0.14 |
| Isiolo | 0.01 | 0.18 | 0.03 | 0.08 |
| Kajiado | 0.01 | 0.20 | 0.02 | 0.12 |
| Kakamega | 0.03 | 0.24 | 0.02 | 0.08 |
| Elgeyo-Marakwet | 0.01 | 0.20 | 0.02 | 0.12 |
| Kericho | 0.01 | 0.20 | 0.02 | 0.12 |
| Kiambu | 0.02 | 0.27 | 0.03 | 0.10 |
| Kilifi | 0.00 | 0.23 | 0.03 | 0.12 |
| Kirinyaga | 0.02 | 0.27 | 0.03 | 0.10 |
| Kisii | 0.01 | 0.26 | 0.03 | 0.14 |
| Kisumu | 0.01 | 0.26 | 0.03 | 0.14 |
| Kitui | 0.01 | 0.18 | 0.03 | 0.08 |
| Kwale | 0.00 | 0.23 | 0.03 | 0.12 |
| Laikipia | 0.01 | 0.20 | 0.02 | 0.12 |
| Lamu | 0.00 | 0.23 | 0.03 | 0.12 |
| Machakos | 0.01 | 0.18 | 0.03 | 0.08 |
| Makueni | 0.01 | 0.18 | 0.03 | 0.08 |
| Mandera | 0.01 | 0.02 | 0.00 | 0.01 |
| Marsabit | 0.01 | 0.18 | 0.03 | 0.08 |
| Meru | 0.01 | 0.18 | 0.03 | 0.08 |
| Migori | 0.01 | 0.26 | 0.03 | 0.14 |
| Murang'a | 0.02 | 0.27 | 0.03 | 0.10 |
| Nakuru | 0.01 | 0.20 | 0.02 | 0.12 |
| Nandi | 0.01 | 0.20 | 0.02 | 0.12 |
| Narok | 0.01 | 0.20 | 0.02 | 0.12 |
| Nyamira | 0.01 | 0.26 | 0.03 | 0.14 |
| Nyandarua | 0.02 | 0.27 | 0.03 | 0.10 |
| Nyeri | 0.02 | 0.27 | 0.03 | 0.10 |
| Samburu | 0.01 | 0.20 | 0.02 | 0.12 |
| Siaya | 0.01 | 0.26 | 0.03 | 0.14 |
| Taita Taveta | 0.00 | 0.23 | 0.03 | 0.12 |
| Tana River | 0.00 | 0.23 | 0.03 | 0.12 |
| Tharaka | 0.01 | 0.18 | 0.03 | 0.08 |
| Trans Nzoia | 0.01 | 0.20 | 0.02 | 0.12 |
| Turkana | 0.01 | 0.20 | 0.02 | 0.12 |
| Uasin Gishu | 0.01 | 0.20 | 0.02 | 0.12 |
| Vihiga | 0.03 | 0.24 | 0.02 | 0.08 |
| Wajir | 0.01 | 0.02 | 0.00 | 0.01 |
| West Pokot | 0.01 | 0.20 | 0.02 | 0.12 |

Table S17. Risk groups by city taken from the DHS 2008/2009.

| City | Proportion of Men Risk Group 1 (Reporting Commercial Sex) | Proportion of Men Risk Group 2 (Reporting Casual Sex) | Proportion of Women Risk Group 1 (Reporting Commercial Sex) | Proportion of Women Risk Group 2 (Reporting Casual Sex) |
| --- | --- | --- | --- | --- |
| Nairobi | 0.01 | 0.33 | 0.01 | 0.20 |
| Mombasa | 0.00 | 0.23 | 0.03 | 0.16 |
| Kisumu | 0.05 | 0.25 | 0.01 | 0.20 |

Table S18 and Table S19 give those parameters which describe the MSM. Whilst only low risk bisexual MSM are included in the county models (Table S18), all three risk groups are included in city models, as a result estimates are needed of the proportion of MSM who are low risk bisexual and the proportion of high risk MSM who are exclusive (Table S19). These estimates are taken from the MARPs geographic mapping report [8]. Key studies which were used to inform each parameter are referenced.

Table S18. MSM parameters across all county models.

| County | Proportion of Men who are MSM | Proportion of MSM who are Bisexual |
| --- | --- | --- |
| All Counties | 0.03 [27] | 1 |

Table S19. MSM parameters for city models.

| City | Proportion of Men who are MSM | Proportion of MSM who are low risk and bisexual | Proportion of high risk MSM who are exclusive (do not have sex with women) | HIV prevalence among high risk MSM | Reference year for the HIV prevalence among high risk MSM | HIV prevalence among low risk MSM |
| --- | --- | --- | --- | --- | --- | --- |
| Nairobi | 0.03 [27] | 0.95 [8] | 0.78 [8] | 40.00 [28] | 2010 | 10.60 [29] |
| Mombasa | 0.03 [27] | 0.91 [8] | 0.59 [8] | 25.00 [30] | 2007 |  |
| Kisumu | 0.03 [27] | 0.47 [8] | 0.33 [8] | 20.00 (based on [31] but adjusted to account for age structure of the sample) | 2010 |  |

Table S20 and Table S21 describe the estimated male circumcision and ART coverage levels by county or city respectively.

Table S20. Existing interventions by county for the year 2011 (taken from Kenya County Fact Sheets). Note that ART coverage was capped in the model at 90%, some reported ART coverage levels exceed 1 in the Fact Sheets.

| County | Proportion of Men Circumcised | ART Coverage |
| --- | --- | --- |
| Baringo | 0.98 | 0.25 |
| Bomet | 0.95 | 0.5 |
| Bungoma | 0.96 | 0.96 |
| Busia | 0.51 | 0.69 |
| Embu | 0.98 | 0.81 |
| Garissa | 0.96 | 0.23 |
| Homa Bay | 0.1 | 0.58 |
| Isiolo | 0.97 | 1 |
| Kajiado | 0.93 | 1 |
| Kakamega | 0.95 | 0.73 |
| Elgeyo-Marakwet | 0.83 | 0.31 |
| Kericho | 0.82 | Estimate not given |
| Kiambu | 0.96 | 1.01 |
| Kilifi | 0.99 | 0.94 |
| Kirinyaga | 0.97 | 0.77 |
| Kisii | 0.99 | 0.45 |
| Kisumu | 0.27 | 1.09 |
| Kitui | 0.97 | 0.66 |
| Kwale | 0.99 | 0.3 |
| Laikipia | 0.89 | 0.35 |
| Lamu | 0.97 | 0.28 |
| Machakos | 1 | 0.81 |
| Makueni | 1 | 0.53 |
| Mandera | 0.99 | 0.04 |
| Marsabit | 1 | 0.52 |
| Meru | 0.92 | 0.65 |
| Migori | 0.42 | 0.7 |
| Murang'a | 0.93 | 0.43 |
| Nakuru | 0.92 | 0.66 |
| Nandi | 0.91 | 0.63 |
| Narok | 0.95 | 0.36 |
| Nyamira | 0.98 | 0.85 |
| Nyandarua | 0.92 | 0.8 |
| Nyeri | 0.94 | 1.01 |
| Samburu | 0.69 | 0.13 |
| Siaya | 0.82 | 0.82 |
| Taita Taveta | 0.94 | 0.1 |
| Tana River | 0.98 | 0.49 |
| Tharaka | 0.91 | 0.83 |
| Trans Nzoia | 0.92 | 0.39 |
| Turkana | 0.82 | 0.12 |
| Uasin Gishu | 0.89 | 4 |
| Vihiga | 0.98 | 0.76 |
| Wajir | 0.98 | 0.34 |
| West Pokot | 0.97 | 0.43 |

Table S21. Existing interventions by city for the year 2011 (also taken from the County Factsheets). Note that ART coverage was capped in the model at 90%.

| City | Proportion of Men Circumcised | ART Coverage |
| --- | --- | --- |
| Nairobi | 0.85 | 0.87 |
| Mombasa | 0.82 | 0.79 |
| Kisumu | 0.27 | 1.09 |

## 9 Methods for the Interpolated Prevalence Maps

Interpolated prevalence surfaces are used to inform the spatiotemporal trend in prevalence. The 2008/2009 DHS data was used to examine spatial trends in HIV prevalence [10]. Although the DHS provides good geographical coverage with numerous samples from across Kenya, geo-referenced data is available only for a limited number of years, so it is not possible to use this data to assess the change in the prevalence over time. To inform the time trends in prevalence we used the antenatal clinic data recorded annually from 1990 [25].

### DHS 2008/2009

The HIV prevalence in each location in 2008 was estimated based on an interpolated map produced using the 2008/2009 DHS data [10]. Each of the sampling clusters from across the country were geocoded, and are plotted on the map of Kenya. Those clusters with missing data on latitude and longitude, or in which no one was tested, were removed from the data. As clusters typically only include a small number of individuals tested for HIV, much of the variation between sample points could be due to noise rather than real differences in prevalence between locations. As such methods used to examine the trend in prevalence across regions must account for this. Larmarange et al compare a number of different methods in their ability to predict regional HIV prevalence trends from a ‘simulated’ DHS sample in a fictional country [32]. The best method was found to be a Kernel estimator approach with adaptive bandwidths such that there are an equal number of individuals sampled. This method is used here to produce HIV prevalence surfaces. Data was weighted using the HIV weighting variable which takes into account HIV testing non-response in addition to the sampling frame used to identify households for inclusion in the survey. Prevalence within each county boundary was taken from the interpolated map to produce the 2008 county specific estimates of prevalence.

### Sentinel Antenatal Clinic Data

Sentinel Antenatal Clinic data is available for the years 1990-2011 [25]. The ANC sites were plotted using the official latitude and longitude coordinates and interpolation between prevalence estimates from each site performed. These maps were used to produce a time trend of prevalence for use in the model fitting process. Inverse Distance Weighting was used to produce a smoothed prevalence surface between measured prevalence at ANC sites [33]. Where data was insufficient to cover the whole map, ANC data from neighbouring countries was used. To avoid bias from selective inclusion of data points, all official sentinel surveillance data from all 5 countries neighbouring Kenya (Uganda, Tanzania, Ethiopia, South Sudan and Somalia) was utilised in the production of the ANC maps [34]. Antenatal clinic sites were geocoded using the National Geospatial-Intelligence Agency GEOnet Names Server and spatialepidemiology.net [35, 36]. The majority of clinics were geocoded either specifically to the clinic or at town or village level, where the location could not be verified the lowest regional-level geocode was used or the data excluded if the location insufficiently specific. Where data was missing from a neighbouring country for one of the years included in the ANC mapping (e.g. due to alternating surveillance years), data from a year either side of the missing year could be substituted in for that country.

**References**

1. Anderson SJ, Cherutich P, Kilonzo N, Cremin I, Fecht D, Kimanga D, et al. Maximising the effect of combination HIV prevention through prioritisation of the people and places in greatest need: a modelling study. Lancet. 2014;384(9939):249-56.

2. Hallett TB, Baeten JM, Heffron R, Barnabas R, de Bruyn G, Cremin I, et al. Optimal uses of antiretrovirals for prevention in HIV-1 serodiscordant heterosexual couples in South Africa: a modelling study. PLoS Med. 2011;8(11):e1001123.

3. GARNETT GP, ANDERSON RM. Balancing sexual partnership in an age and activity stratified model of HIV transmission in heterosexual populations. Mathematical Medicine and Biology. 1994;11(3):161-92.

4. Hallett TB, Gregson S, Mugurungi O, Gonese E, Garnett GP. Assessing evidence for behaviour change affecting the course of HIV epidemics: a new mathematical modelling approach and application to data from Zimbabwe. Epidemics. 2009;1:108-17.

5. Lodi S, Phillips A, Touloumi G, Geskus R, Meyer L, Thiebaut R, et al. Time From Human Immunodeficiency Virus Seroconversion to Reaching CD4+ Cell Count Thresholds < 200, < 350, and < 500 Cells/mm(3): Assessment of Need Following Changes in Treatment Guidelines. Clinical Infectious Diseases. 2011;53(8):817-25.

6. Hollingsworth TD, Anderson RM, Fraser C. HIV-1 Transmission, by Stage of Infection. Journal of Infectious Diseases. 2008;198(5):687-93.

7. National AIDS and STI Control Programme (NASCOP). Kenya, County HIV Service Delivery Profiles. 2013.

8. National AIDS and STI Control Programme (NASCOP) & National AIDS Control Council (NACC) . Geographic Mapping of Most at Risk Populations for HIV (MARPs) in Kenya. 2012.

9. The World Bank. Population growth (annual %). Available from: <http://data.worldbank.org/indicator/SP.POP.GROW>.

10. Kenya National Bureau of Statistics (KNBS) and ICF Macro. Kenya Demographic and Health Survey 2008-2009. Calverton, Maryland; 2010.

11. National AIDS and STI Control Programme (NASCOP) & National AIDS Control Council (NACC). Kenya AIDS Epidemic update 2011. Nairobi, Kenya; 2012.

12. Auvert B, Taljaard D, Lagarde E, Sobngwi-Tambekou J, Sitta R, Puren A. Randomized, controlled intervention trial of male circumcision for reduction of HIV infection risk: the ANRS 1265 Trial. PLoS Med. 2005;2(11):e298.

13. Cremin I, Alsallaq R, Dybul M, Piot P, Garnett G, Hallett TB. The new role of antiretrovirals in combination HIV prevention: a mathematical modelling analysis. AIDS (London, England). 2013;27(3):447-58.

14. Schwartländer B, Stover J, Hallett T, Atun R, Avila C, Gouws E, et al. Towards an improved investment approach for an effective response to HIV/AIDS. The Lancet. 2011;377(9782):2031-41.

15. Rethink HIV. Smarter Ways to Invest in Ending HIV in Sub-Saharan Africa. Lomborg B, editor 2012.

16. Cherutich P, Athanasius, Ochieng, Kimanga D, Mwandi Z, Mwalili S, et al. Progress in voluntary medical male circumcision service provision - Kenya, 2008-2011. Morbidity and Mortality Weekly Report. 2012;61(47).

17. Bailey RC, Moses S, Parker CB, Agot K, Maclean I, Krieger JN, et al. Male circumcision for HIV prevention in young men in Kisumu, Kenya: a randomised controlled trial. The Lancet.369(9562):643-56.

18. Cohen MS, Chen YQ, McCauley M, Gamble T, Hosseinipour MC, Kumarasamy N, et al. Prevention of HIV-1 Infection with Early Antiretroviral Therapy. New England Journal of Medicine. 2011;365(6):493-505.

19. Menzies NA, Berruti AA, Berzon R, Filler S, Ferris R, Ellerbrock TV, et al. The cost of providing comprehensive HIV treatment in PEPFAR-supported programs. Aids. 2011;25(14):1753-60.

20. WHO, UNICEF, UNAIDS. Global Update on HIV Treatment 2013: Results, impact and opportunities.; 2013.

21. The U.S. President’s Emergency Plan for AIDS Relief (PEPFAR) . PEPFAR Bluepint: Creating an AIDS-free Generation.; 2012 November 2012.

22. Hallett TB, Aberle-Grasse J, Bello G, Boulos LM, Cayemittes MP, Cheluget B, et al. Declines in HIV prevalence can be associated with changing sexual behaviour in Uganda, urban Kenya, Zimbabwe, and urban Haiti. Sexually transmitted infections. 2006;82 Suppl 1:i1-8.

23. Donnell D, Baeten JM, Kiarie J, Thomas KK, Stevens W, Cohen CR, et al. Heterosexual HIV-1 transmission after initiation of antiretroviral therapy: a prospective cohort analysis. The Lancet. 2010;375(9731):2092-8.

24. Gray RH, Kigozi G, Serwadda D, Makumbi F, Watya S, Nalugoda F, et al. Male circumcision for HIV prevention in men in Rakai, Uganda: a randomised trial. The Lancet.369(9562):657-66.

25. National AIDS and STI Control Programme (NASCOP). Sentinel surveillance for HIV and Syphilis infection among pregnant women from antenatal clinics in Kenya. Nairobi, Kenya; 2011.

26. Kenya Open Data. Kenya Open Data 2011. Available from: <https://opendata.go.ke/>.

27. Caceres CF, Konda K, Segura ER, Lyerla R. Epidemiology of male same-sex behaviour and associated sexual health indicators in low- and middle-income countries: 2003-2007 estimates. Sexually transmitted infections. 2008;84 Suppl 1:i49-i56.

28. McKinnon LR, Gakii G, Juno JA, Izulla P, Munyao J, Ireri N, et al. High HIV risk in a cohort of male sex workers from Nairobi, Kenya. Sexually transmitted infections. 2014;90(3):237-42.

29. Angala P, Parkinson A, Kilonzo N, Natecho A, M T, editors. Men who have sex with men (MSM) as presented in VCT data in Kenya. AIDS 2006-XVI International AIDS Conference; 2006; Toronto, ON, Canada.

30. Sanders EJ, Graham SM, Okuku HS, van der Elst EM, Muhaari A, Davies A, et al. HIV-1 infection in high risk men who have sex with men in Mombasa, Kenya. Aids. 2007;21(18):2513-20.

31. National AIDS and STI Control Programme (NASCOP). MARPs Surveillance Report 2012. 2012.

32. Larmarange J, Vallo R, Yaro S, Msellati P, Méda N. Methods for mapping regional trends of HIV prevalence from Demographic and Health Surveys (DHS) Cybergeo : European Journal of Geography [Internet]. 2012 20/11/12. Available from: <http://cybergeo.revues.org/24606>.

33. Shepard D, editor A two dimensional interpolation function for irregularly-spaced data. Proceedings of the 1968 ACM National Conference; 1968; ACM, New York, NY, USA: ACM.

34. Joint United Nations Programme on HIV/ AIDS (UNAIDS). National HIV estimates file 2013 4 December 2015 Available from: <http://apps.unaids.org/spectrum/>.

35. Aanensen D. spatialepidemiology.net Available from: <http://www.spatialepidemiology.net/>.

36. National Geospatial Intelligence Agency. NGA GEOnet Names Server (GNS) 2013 Available from: <http://geonames.nga.mil/gns/html/>.
